# Dual-Band Electrochromic Poly(Amide-Imide)s with Redox-Stable *N*,*N*,*N*’,*N*’-Tetraphenyl-1,4-Phenylenediamine Segments

**DOI:** 10.3390/polym18010139

**Published:** 2026-01-03

**Authors:** Bo-Wei Huang, Sheng-Huei Hsiao

**Affiliations:** Department of Chemical Engineering and Biotechnology, National Taipei University of Technology, Taipei 10608, Taiwan; s0979121791@gmail.com

**Keywords:** triphenylamine, poly(amide-imide)s, electrochemistry, electrochromism, redox-active polymers

## Abstract

Two amide-preformed aromatic diamine monomers, *N*,*N*-bis(4-(3-aminobenzamido)phenyl)-*N*’,*N*’-bis(4-methoxyphenyl)-1,4-phenylenediamine (*m*-**6**) and *N*,*N*-bis(4-(4-aminobenzamido)phenyl)-*N*’,*N*’-bis(4-methoxyphenyl)-1,4-phenylenediamine (*p*-**6**), were synthesized and utilized to prepare two series of electroactive poly(amide-imide)s (PAIs) through a two-step polycondensation reaction with commercially available aromatic tetracarboxylic dianhydrides. The obtained polymers exhibited solubility in various polar organic solvents, and most of them could form transparent, flexible films via solution casting. Thermal analysis indicated glass transition temperatures (*T*_g_) ranging from 250 °C to 277 °C, as measured by DSC, with no significant weight loss observed before 400 °C in TGA tests. Cyclic voltammograms (CV) of the polymer films on ITO-coated glass substrates revealed two reversible oxidation redox pairs between 0.67 and 1.04 V vs. Ag/AgCl in an electrolyte-containing acetonitrile solution. The PAI films showed stable redox activity with high optical contrast both in the visible and near-infrared regions, transitioning from colorless in the neutral state to green and blue in the oxidized states. Furthermore, the polymer films retained good electrochemical and electrochromic stability even after more than 100 cyclic switching operations. The PAIs displayed outstanding electrochromic performance, including high optical contrast (up to 95%), rapid response times (below 4.6 s for coloring and 5.7 s for bleaching), high coloration efficiency (up to 240 cm^2^/C), and low decay in optical contrast (less than 5% after 100 switching cycles for most PAIs).

## 1. Introduction

Electrochromism is a process that allows for the reversible change in color or light transmittance of a material through the application of a small voltage [1]. Due to their low power consumption and controllable color changes, electrochromic materials have garnered significant attention in recent years [2]. These materials are utilized in a variety of applications, including smart windows, anti-glare rearview mirrors, displays, eyewear, wearable electronics, adaptive camouflage, energy storage systems, and other versatile electro-optical devices [3,4,5,6,7,8,9]. A notable example is the dimmable windows used in the Boeing 787 Dreamliner.

Electrochromic materials can be categorized into several types, including metal oxides (such as WO_3_ and NiO_x_) [10], metal complexes [11], organic small molecules (like viologens) [12], and conjugated polymers [13]. Among these, conjugated polymers are extensively studied due to their numerous advantages, including fast response times, high coloration efficiency, easy color tuning, and the capability to produce flexible devices using simple solution-processing techniques [14,15]. In recent years, many researchers have focused on designing polymer structures by incorporating electron-donating and electron-accepting units to optimize color and performance. These materials can exhibit multiple color states, absorb light across both the visible and near-infrared regions, and maintain stability after repeated switching. As a result, organic electrochromic polymers are considered promising candidates for future smart and energy-efficient devices [16].

Triarylamine derivatives are well-known for their photoactive and reversible oxidation behavior, making them suitable for various optoelectronic applications, including photoconductors, hole transport layers, and light-emitting devices [17,18]. These compounds can be easily oxidized to form stable radical cations, a process that is always accompanied by a noticeable color change. Since 2005, Liou’s research team has pioneered investigations into the intriguing electrochromic properties of high-performance polymers, such as aromatic polyamides and polyimides, which incorporate triphenylamine (TPA) units as electrochromic chromophores [19,20]. Numerous TPA-based electrochromic polymers have been reported in the literature [21,22,23,24,25,26]. For example, *N*,*N*,*N*’,*N*’*-*tetraphenyl-1,4-phenylenediamine (TPPA), also known as bis(triphenylamine), contains two adjacent TPA redox-active centers linked by a 1,4-phenylene bridge. The cation radicals of TPPA are classified as symmetrical delocalized Class III mixed-valence systems, characterized by strong electronic coupling [27,28,29,30]. The unique structural features and robust intervalence charge transfer (IVCT) effect of TPPA endow TPPA-based polymers with the ability to exhibit multicolor states and significant near-infrared (NIR) activity [31,32,33,34,35,36]. As a result, TPA-based polymers that incorporate two or more electroactive nitrogen centers typically demonstrate distinct IVCT absorption in the NIR region [37,38,39,40,41,42], which is advantageous for applications such as smart windows and energy-efficient building technologies [43].

It has been reported that triarylamine-based polyimides generally exhibit inferior electrochemical and electrochromic stability compared to their polyamide counterparts, primarily due to the strong electron-withdrawing nature of the imide groups [44,45]. These groups tend to increase the oxidation potential of the triarylamine unit while reducing the stability of the resulting radical cation after oxidation. To address this issue, previous studies have introduced spacers between the triarylamine core and the imide ring to minimize the electronic influence of the imide group, thereby enhancing the overall electrochemical and optical performance of these electroactive polymers [46,47,48]. In this work, a set of isomeric TPPA-based diamide–diamine monomers, *N*,*N*-bis(4-(3-(or 4-)aminobenzamido)-phenyl)-*N*’,*N*’-bis(4-methoxyphenyl)-1,4-phenylenediamine (*m*-**6** and *p*-**6**), will be synthesized and subsequently polymerized with various aromatic dianhydrides to produce two series of poly(amide-imide)s (PAIs) containing TPPA and benzamide units in the PAI backbones. The incorporation of a benzamide spacer is anticipated to effectively mitigate the electron-withdrawing effects of the imide group, resulting in PAI materials with balanced thermal stability and solution processability, along with enhanced electrochemical and electrochromic properties that exhibit reversible multistage redox transitions.

## 2. Materials and Methods

### 2.1. Materials

The following chemicals were used as received from local suppliers: *p*-fluoronitrobenzene (Acros, Geel, Belgium), *p*-anisidine (Acros, Geel, Belgium), dimethyl sulfoxide (DMSO, Tedia, Fairfield, OH, USA), copper(I) iodide (CuI, Thermo, Waltham, MA, USA), 18-crown-6 ether (TCI, Tokyo, Japan), 1,2-dichlorobenzene (Thermo, Waltham, MA, USA), potassium carbonate (K_2_CO_3_, Duksan, Ansan-si, Korea), hydrazine monohydrate (Alpha, Ward Hill, MA, USA), 10% palladium on activated carbon (Pd/C, Fluka, Seelze, Germany), 3-nitrobenzoyl chloride (Acros, Geel, Belgium), 4-nitrobenzoyl chloride (Acros, Geel, Belgium), 4-nitroaniline (Acros, Geel, Belgium), 4-iodoanisole (Thermo, Waltham, MA, USA), and cesium fluoride (CsF, Acros, Geel, Belgium). The following organic solvents were dried over calcium hydride for 24 h, distilled under reduced pressure, and stored over 4 Å molecular sieves: *N*-methyl-2-pyrrolidone (NMP, Tedia, Fairfield, OH, USA), *N*,*N*-dimethylformamide (DMF, Avantor, Radnor, PA, USA), *N*,*N*-dimethylacetamide (DMAc, Duksan, Ansan-si, Republic of Korea), and pyridine. Commercially available aromatic tetracarboxylic dianhydrides, including pyromellitic dianhydride (PMDA, **7a**, TCI, Tokyo, Japan), 3,3′,4,4′-biphenyltetracarboxylic dianhydride (BPDA, **7b**, Oxychem), 4,4′-oxydiphthalic anhydride (ODPA, **7c**, Oxychem, Dallas, TX, USA), 4,4′-(hexafluoroisopropylidene)diphthalic anhydride (6FDA, **7d**, Oxychem, Dallas, TX, USA), and 3,3′,4,4′-diphenylsulfonetetracarboxylic dianhydride (DSDA, **7e**, Oxychem, Dallas, TX, USA), were purified by dehydration at 250 °C for 5 h.

**Scheme 1:**
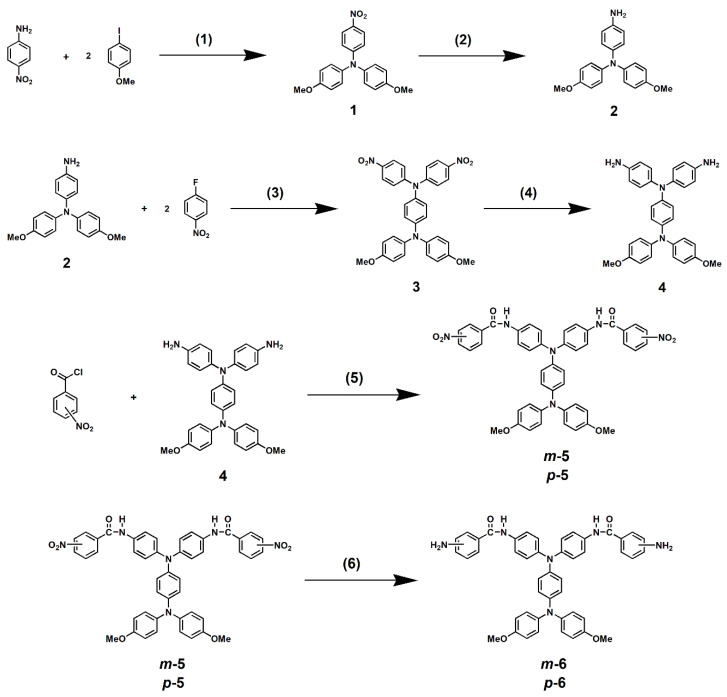
Synthetic route of diamide–diamine monomers *m*-**6** and *p*-**6**: (1) CuI, 18-crown-6, K_2_CO_3_, 1,2-dichlorobenzene, 180 °C, 24 h; (2) hydrazine, Pd/C, ethanol/THF, 80 °C, 4 h; (3) CsF, DMSO, 120 °C, 1 day; (4) hydrazine, Pd/C, ethanol/THF, 80 °C, 4 h; (5) *m*-nitrobenzoyl chloride or *p*-nitrobenzoyl chloride, pyridine, DMF, 0 °C, 4 h; (6) hydrazine, Pd/C, ethanol/THF, 90 °C, 24 h.

Following the synthetic procedure reported previously [31], *N*,*N*-bis(4-aminophenyl)-*N*’,*N*’-bis(4-methoxyphenyl)-1,4*-*phenylenediamine (**4**) was synthesized via a four-step reaction sequence (Scheme 1). First, 4,4′-dimethoxy-4″-nitrotriphenylamine **(1)** was synthesized via a copper-catalyzed Ullmann coupling reaction using *p*-nitroaniline and *p*-anisidine as the starting materials. The reaction was performed in 1,2-dichlorobenzene with CuI, 18-crown-6, and K_2_CO_3_ at 180 °C for 24 h. The resulting nitro compound was then reduced with hydrazine monohydrate in the presence of Pd/C catalyst to give 4-amino-4′,4″-dimethoxytriphenylamine **(2)**. Subsequently, compound **(2)** underwent a nucleophilic aromatic substitution reaction with *p*-fluoronitrobenzene in DMSO using CsF as the base, affording *N*,*N*-bis(4-methoxyphenyl)-*N*’,*N*’-bis(4-nitrophenyl)-1,4-phenylenediamine **(3)**. This dinitro compound was further reduced with hydrazine monohydrate and Pd/C in ethanol/THF under reflux conditions to give the TPPA-diamine **4**.

### 2.2. Synthesis of N,N-Bis(4-(3-Nitrobenzamido)Phenyl)-N’,N’-Bis(4-Methoxyphenyl)- 1,4-Phenylenediamine (m-***5***)

**Figure 1:**
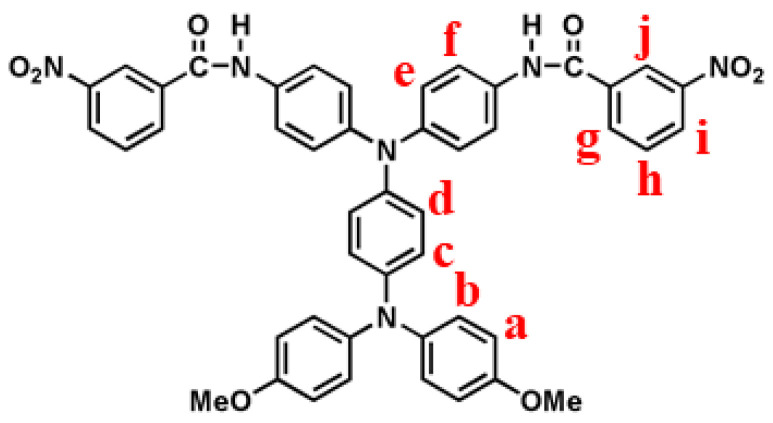
Molecular structure of compound *m*-**5**.

In a 250 mL three-neck round-bottom flask immersed in an ice bath, 2.00 g (3.9 mmol) of TPPA-diamine **4** and 2 mL of pyridine were added to 20 mL of DMF. A solution of 1.62 g (8.8 mmol) of 3-nitrobenzoyl chloride in 3 mL of DMF was then gradually added to the reaction mixture under continuous stirring to form the amide product. The mixture was stirred at 0 °C for 2 h and then at room temperature for 3 h. Upon completion of the reaction, the mixture was poured into a solution of methanol and water (300 mL, 2:1 by volume) to induce precipitation. The resulting solid product was collected by filtration. After drying, 2.04 g (65% yield) of the diamide–dinitro compound *m*-**5** was obtained as a brown powder with a melting point (mp) of 205–207 °C. FT-IR (KBr): 3243 cm^−1^ (amide N–H stretch), 1650 cm^−1^ (amide C=O stretch), 1504, 1309 cm^−1^ (–NO_2_ stretch). ^1^H NMR (600 MHz, DMSO-*d*_6_, δ, ppm) (Assignments of aromatic protons refer to Figure 1): 3.73 (s, 6H, –OMe), 6.80 (d, *J* = 9.0 Hz, 2H, Hc), 6.89 (d, *J* = 9.0 Hz, 4H, Hb), 6.92 (d, *J* = 9.0 Hz, 4H, Hd), 6.89–7.03 (m, 8H, Ha + He), 7.69 (d, *J* = 8.9 Hz, 4H, Hf), 7.84 (t, *J* = 8.0 Hz, 2H, Hh), 8.42 (dd, *J* = 8.2, 2.3 Hz, 2H, Hi), 8.44 (dd, *J* = 8.2, 2.3 Hz, 2H, Hg), 8.78 (t, *J* = 1.9 Hz, 2H, Hj), 10.53 (s, 2H, amide N–H). HRMS (*m*/*z*) Found: 800.2600 (M^+^), 801.2632 (MH^+^); Exact mass: 800.2597 for molecular formula C_46_H_36_N_6_O_8_. 

### 2.3. Synthesis of N,N-Bis(4-(4-Nitrobenzamido)Phenyl)-N’,N’-Bis(4-Methoxyphenyl)- 1,4-Phenylenediamine (p-***5***)

**Figure 2:**
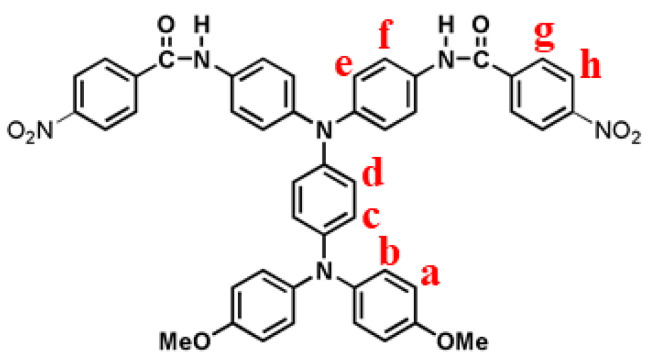
Molecular structure of compound *p*-**5**.

In a 250 mL three-neck round-bottom flask immersed in an ice bath, 1.00 g (2.0 mmol) of TPPA-diamine **4** and 1.5 mL of pyridine were added to 40 mL of DMF. Subsequently, a solution of 0.81 g (4.4 mmol) of 4-nitrobenzoyl chloride in 3 mL of DMF was added to the reaction mixture. The mixture was stirred under a nitrogen atmosphere at room temperature for 3 h to ensure complete reaction. Upon completion of the reaction, the mixture was poured into a solution of methanol and water (300 mL, 2:1 by volume) to induce precipitation. The resulting solid product was collected by filtration and purified by recrystallization from ethanol. After drying, 1.07 g (67% yield) of the diamide–dinitro compound *p*-**5** was obtained as a purplish-red powder; mp = 181–183 °C. FT-IR (KBr): 3252 cm^−1^ (amide N–H stretch), 1650 cm^−1^ (amide C=O stretch), 1503, 1312 cm^−1^ (–NO_2_ stretch). ^1^H NMR (600 MHz, DMSO-*d*_6_, δ, ppm) (Assignments of aromatic protons refer to Figure 2): 3.72 (s, 6H, –OMe), 6.80 (d, *J* = 9.0 Hz, 2H, Hc), 6.85–6.95 (m, 6H, Hb + Hd), 7.00 (d, *J* = 8.9 Hz, 8H, Ha + He), 7.69 (d, *J* = 8.9 Hz, 4H, Hf), 8.17 (d, *J* = 8.9 Hz, 4H, Hg), 8.36 (d, *J* = 8.9 Hz, 4H, Hh), 10.51 (s, 2H, amide N–H). HRMS (*m*/*z*) Found: 800.2578 (M^+^), 801.2617 (MH^+^); Exact mass: 800.2597 for molecular formula C_46_H_36_N_6_O_8_.

### 2.4. Synthesis of N,N-Bis(4-(3-Aminobenzamido)Phenyl)-N’,N’-Bis(4-Methoxyphenyl)- 1,4-Phenylenediamine (m-***6***)

**Figure 3:**
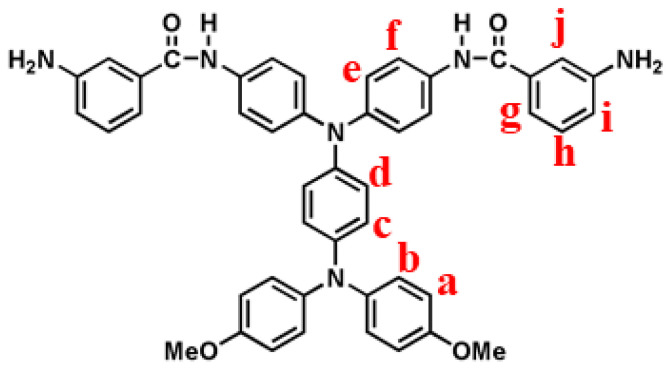
Molecular structure of compound *m*-**6**.

In a 250 mL three-neck round-bottom flask equipped with a magnetic stir bar, 1.00 g of the diamide–dinitro compound *m*-**5** was dissolved in a mixture of 150 mL of ethanol and 50 mL of THF. After complete dissolution, 0.1 g of Pd/C and 0.1 mL of hydrazine were added sequentially. The reaction mixture was then refluxed at 90 °C for 4 h. Upon completion of the reaction, the mixture was hot-filtered to remove the Pd/C catalyst. The filtrate was concentrated in a rotatory evaporator and cooled to precipitate the product, which was collected by filtration and dried in vacuo at 50 °C, yielding 0.92 g (99%) of a green powder with a melting point of 148–150 °C. FT-IR (KBr): 3443, 3339 cm^−1^ (–NH_2_ stretch). ^1^H NMR (600 MHz, DMSO-*d*_6_, δ, ppm) (Assignments of aromatic protons refer to Figure 3): 3.72 ppm (s, 6H, –OMe), 5.28 ppm (s, 4H, –NH_2_), 6.73 (d, *J* = 8.0 Hz, 2H, Hi), 6.78 (d, *J* = 9.0 Hz, 2H, Hc), 6.88 (d, *J* = 8.9 Hz, 6H, Hb + Hd), 6.97 (d, *J* = 8.9 Hz, 4H, He), 6.99 (d, *J* = 8.9 Hz, 4H, Ha), 7.05 (d, *J* = 8.0 Hz, 2H, Hg), 7.07 (s, 2H, Hj), 7.13 (t, *J* = 7.7 Hz, 2H, Hh), 7.66 (d, 4H, *J* = 8.9 Hz, Hf), 9.99 (s, 2H, amide N–H). HRMS (*m*/*z*) Found: 740.3121 (M^+^), 741.3134 (MH^+^); Exact mass: 740.3114 for molecular formula C_46_H_40_N_6_O_4_.

### 2.5. Synthesis of N,N-Bis(4-(4-Aminobenzamido)Phenyl)-N’,N’-Bis(4-Methoxyphenyl)- 1,4-Phenylenediamine (p-***6***)

**Figure 4:**
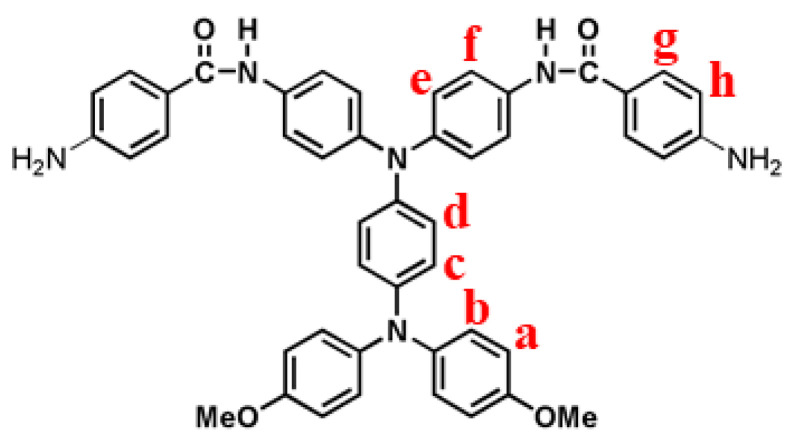
Molecular structure of compound *p*-**6**.

In a 250 mL three-neck round-bottom flask equipped with a magnetic stir bar, 1.00 g of the diamide–dinitro compound *p*-**5** was dissolved in a mixture of 150 mL ethanol and 50 mL THF. After complete dissolution, 0.1 g of Pd/C and 0.1 mL of hydrazine were added sequentially. The reaction mixture was then refluxed at 90 °C for one day. Upon completion of the reaction, the mixture was hot-filtered to remove the Pd/C catalyst. The filtrate was concentrated in a rotatory evaporator and cooled to precipitate the product, which was collected by filtration and dried in vacuo at 50 °C, yielding 0.91 g (98%) of a green powder with a melting point of 143–145 °C. FT-IR (KBr): 3465, 3356 cm^−1^ (–NH_2_ stretch). ^1^H NMR (600 MHz, DMSO-*d*_6_, δ, ppm) (Assignments of aromatic protons refer to Figure 4): 3.72 (s, 6H, –OMe), 5.71 (s, 4H, –NH_2_), 6.59 (d, *J* = 8.6 Hz, 4H, Hh), 6.78 (d, *J* = 8.9 Hz, 2H, Hc), 6.87 (d, *J* = 8.9 Hz, 2H, Hd), 6.89 (d, *J* = 9.0 Hz, 4H, Hb), 6.94 (d, *J* = 8.6 Hz, 4H, Hf), 6.98 (d, *J* = 9.0 Hz, 4H, Ha), 7.64 (d, *J* = 8.9 Hz, 4H, Hf), 7.69 (d, *J* = 8.6 Hz, 4H, Hg), 9.69 (s, 2H, amide N–H). HRMS (*m*/*z*) Found: 740.3123 (M^+^), 741.3136 (MH^+^); Exact mass: 740.3114 for molecular formula C_46_H_40_N_6_O_4_.

### 2.6. Synthesis of Poly(Amide-Imide)s

Two series of novel poly(amide-imide)s (PAIs) were synthesized via a conventional two-step procedure consisting of poly(amic acid) formation followed by thermal imidization. The PAIs were prepared from diamide–diamines *m*-**6** and *p*-**6**, respectively, with five aromatic tetracarboxylic dianhydrides including PMDA, BPDA, ODPA, 6FDA, and DSDA (**7a**–**7e**). A typical example for the synthesis of PAI *m*-**8c** is described as follows. A homogeneous solution of 0.11 g (0.164 mmol) of diamide–diamine *m*-**6** and 0.05 g (0.164 mmol) of ODPA (**7c**) in 1.4 mL of anhydrous DMAc was prepared at a solid content of 10 wt% under a nitrogen atmosphere. The mixture was stirred at room temperature for 10 h to afford the poly(amic acid) precursor. The viscous solution was then cast into a 5 cm-diameter Petri dish and thermally cured in a vacuum oven according to the following programmed heating profile: 90 °C for 3 h, 150 °C for 30 min, 200 °C for 30 min, and 250 °C for 1 h. The resulting PAI *m*-**8c** exhibited an inherent viscosity of 0.31 dL/g, as measured on 0.5 g/dL in DMAc at 30 °C. FTIR (thin film): 3365 cm^−1^ (amide N–H stretch), 1780 and 1720 cm^−1^ (imide ring C=O stretch), 1650 cm^−1^ (amide C=O stretch). ^1^H NMR (600 MHz, DMSO-*d*_6_, δ, ppm): 3.70 (methoxy protons), 6.76–8.20 (aromatic protons), 10.29 (amide protons).

### 2.7. Measurements

Fourier-transform infrared (FTIR) spectra were recorded on a Horiba FT-720 FT-IR spectrometer in the range of 4000–400 cm^−1^. Proton nuclear magnetic resonance (^1^H NMR) spectra were obtained on a Bruker Avance III HD 600 MHz NMR spectrometer using DMSO-*d*_6_ as the solvent and tetramethylsilane (TMS) as the internal standard. High-resolution mass spectroscopy (HRMS) was carried out on an AB SCIEX QSTAR XL Electrospray Ionization Quadrupole Time-of-Flight (ESI-Q/TOF) Mass Spectrometer. The inherent viscosities (*η*_inh_) of the polymers were measured at a concentration of 0.5 g/dL in DMAc at 30 °C using a Cannon-Fenske viscometer. Gel permeation chromatography (GPC) analysis was performed on a Waters chromatography unit interfaced with a Waters 2410 refractive index detector, utilizing a concentration of 3 mg/mL. Two Waters 5 μm Styragel HR-2 and HR-4 columns (7.8 mm I.D. × 300 mm) were connected in series, with NMP as the eluent at a flow rate of 0.6 mL/min and a temperature of 50 °C, calibrated with polystyrene standards. Thermogravimetric analysis (TGA) was carried out on a PerkinElmer Pyris 1 TGA instrument using 3–5 mg of polymer film sample under nitrogen and air atmospheres at a heating rate of 20 °C/min and a gas flow rate of 20 cm^3^/min. Glass transition temperatures (*T*_g_) were determined by differential scanning calorimetry (DSC) using a PerkinElmer DSC 4000 under a nitrogen atmosphere at a heating rate of 20 °C/min. The solubility of the polymers was tested by dissolving 10 mg of sample in 1 mL of various organic solvents under stirring at room temperature or by heating to 80 °C if necessary. Electrochemical measurements were carried out on a CH Instruments 750 A electrochemical analyzer using a three-electrode configuration. A polymer-coated indium tin oxide (ITO) glass slide (area ~ 0.8 cm × 2.2 cm) served as the working electrode, a platinum wire was used as the auxiliary electrode, and a homemade Ag/AgCl (KCl sat.) electrode was employed as the reference. The thickness of the polymer film on the ITO glass substrate is approximately 200 ± 20 nm. All potentials were calibrated using ferrocene as an external standard (+0.44 V vs. Ag/AgCl). The electrochromic behavior was monitored by UV–vis spectroscopy using an Agilent 8453 UV–visible photodiode array spectrophotometer and an electrolytic cell with a 1 cm cuvette, equipped with a polymer-coated ITO working electrode, a platinum wire auxiliary electrode, and a Ag/AgCl (KCl sat.) reference electrode. The color coordinates of the electrochromic films were measured with an Admesy Brontes colorimeter.

## 3. Results and Discussion

### 3.1. Monomer Synthesis

Following a well-established synthetic procedure [31], the TPPA-based diamine **4** was synthesized through a four-step reaction sequence, beginning with the copper-catalyzed Ullmann coupling reaction of *p*-nitroaniline and *p*-anisidine, as illustrated in Scheme 1. The melting point, IR spectra, and ^1^H NMR spectra of compound **4** and its precursor intermediates are consistent with previously reported data [31]. TPPA-diamine **4** was subsequently reacted with either 3-nitrobenzoyl chloride or 4-nitrobenzoyl chloride in DMF, using pyridine as the base, resulting in the formation of the diamide–dinitro compounds, *m*-**5** and *p*-**5**. Finally, compounds *m*-**5** and *p*-**5** were reduced to the target TPPA-based diamide–diamine monomers, *m*-**6** and *p*-**6**, using hydrazine and a Pd/C catalyst.

All the synthesized compounds were characterized by FT-IR spectroscopy. The IR spectra of compounds **1** to **4** are summarized in Figure S1 (Supplementary Materials). Compounds **1** and **3** showed characteristic absorptions at 1580/1579 and 1317/1330 cm^−1^, associated with –NO_2_ asymmetric and symmetric stretching vibrations. After reduction, the absorption bands attributed to the nitro groups disappeared; obvious absorption peaks peculiar to primary amine can be found in the IR spectra of compounds **2** and **4**. In addition to the prominent amide absorptions around 1650 and 3240 cm^−1^ (Figure S2), the IR spectra of compounds *m*-**5** and *p*-**5** displayed clear nitro group absorptions, confirming the successful introduction of nitrobenzamido units through the acylation reaction of TPPA-diamine **4** with 3- or 4-nitrobenzoyl chloride. After reduction, the spectra of *m*-**6** and *p*-**6** exhibited characteristic –NH_2_ stretching bands in the range of 3300–3500 cm^−1^. Furthermore, the structures of the diamide–dinitro compounds *m*-**5** and *p*-**5**, as well as the diamide–diamine monomers *m*-**6** and *p*-**6**, were confirmed by high-resolution (600 MHz) proton NMR spectroscopy (Figures S3–S6). All four compounds displayed prominent singlets at 3.72 ppm corresponding to the methoxy protons, and signals between 9.69 and 10.52 ppm were assigned to the amide protons. The aromatic protons resonated in the ranges of 6.70–8.80 ppm for the dinitro compounds and 6.50–7.80 ppm for the diamine compounds. Characteristic signals for the primary amine were clearly observed at 5.28 and 5.71 ppm in the ^1^H NMR spectra of *m*-**6** and *p*-**6**, respectively. Compounds *m*-**5** and *m*-**6** exhibited slightly more complex splitting patterns in the aromatic region compared to their *p*-counterparts, attributed to the less symmetric *meta*-connected benzene ring. The complete assignments for the *meta*-disubstituted compounds were facilitated by the indicated multiplicities, expected chemical shifts, and 2D NMR spectroscopy. In contrast, the splitting patterns for *p*-**5** and *p*-**6** were simpler, allowing a first-order analysis at 600 MHz to sufficiently explain the spectra. Thus, all proton NMR spectra are consistent with the expected molecular structures of the synthesized amide-preformed dinitro and diamine compounds. Additionally, the molecular masses of the dinitro and diamine compounds were confirmed by high-resolution mass spectrometry (HRMS).

### 3.2. Synthesis of Model Compounds

**Figure 5:**
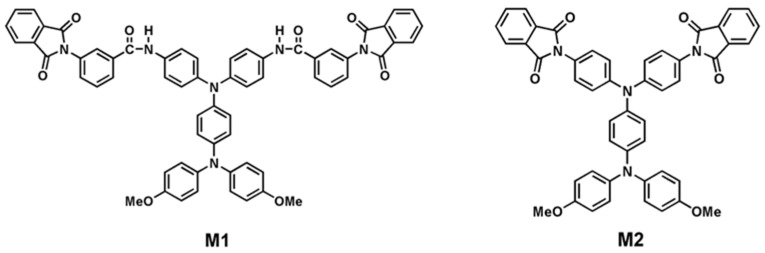
Structures of model compounds **M1** and **M2**.

The model compounds **M1** and **M2** were prepared by reacting diamines *m*-**6** and **4**, respectively, with two equivalents of phthalic anhydride through a one-step dehydration condensation reaction in refluxing glacial acetic acid. The resulting molecular structures are shown in Figure 5. Structural confirmation was achieved using FT-IR spectroscopy (Figure S7), where the disappearance of the –NH_2_ stretching absorption near 3300 cm^−1^ and the emergence of characteristic absorptions at 1781 and 1708 cm^−1^ indicated the successful formation of the imide rings and the consumption of the terminal amino groups.

### 3.3. Polymer Synthesis 

**Scheme 2:**
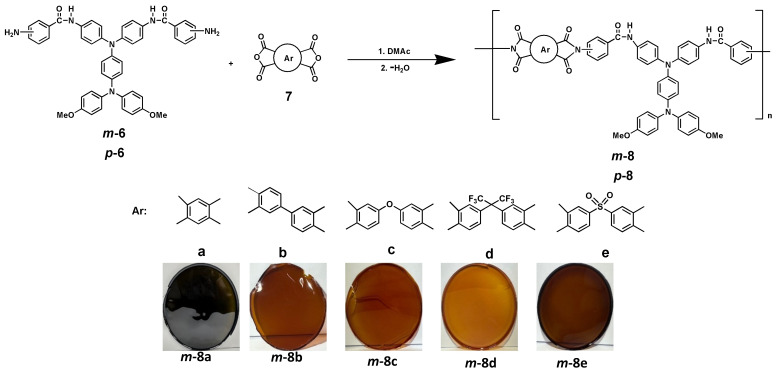
Synthesis of poly(amide-imide)s ***m*-8** and ***p*****-8**.

**Scheme 3:**
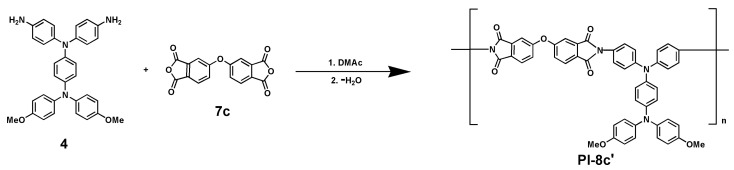
Synthesis of reference polyimide PI-**8c′**.

Two isomeric series of poly(amide-imide)s (PAIs) *m*-**8a**–**8e** and *p*-**8a**–**8e** were synthesized from diamide–diamine monomers *m*-**6** and *p*-**6**, respectively, with five different aromatic dianhydrides (**7a**–**7e**) through a conventional two-step method (Scheme 2). The resulting polymers exhibited inherent viscosities ranging from 0.31 to 0.85 dL/g, as detailed in Table 1, indicating moderate molecular weights suitable for film formation. The GPC data for the PAIs are included in Table S1. All *m*-**8** series PAI films could be cast into continuous films, demonstrating good flexibility and the ability to bend without cracking (see inset in Scheme 2). In contrast, the *p*-**8** series polymers generally produced films with reduced flexibility, likely due to the more extended packing caused by the *para*-linked benzamide units. Nevertheless, all PAIs yielded smooth, well-adhering thin films on the ITO glass substrate, facilitating electrochemical and spectral measurements. For comparison, a polyimide designated as PI-**8c′** was synthesized from TPPA-diamine **4** and ODPA (**7c**) (Scheme 3).

The structures of the poly(amide-imide)s were confirmed using FT-IR and ^1^H NMR spectroscopy. As representative examples, the IR spectra of *m*-**8d** and *p*-**8d** are shown in Figure S8. The absorption bands at 1780 and 1720 cm^−1^ correspond to the asymmetric and symmetric C=O stretching of the imide ring. Additionally, characteristic absorption bands at approximately 1650 cm^−1^ (amide C=O stretching) and 3365 cm^−1^ (amide N–H stretching) are attributed to the amide groups. The typical ^1^H NMR spectra of PAIs *m*-**8d** and *p*-**8d** are presented in Figure S9, further confirming the successful synthesis and structural integrity of the target polymers. The signals at 10.29 ppm indicate the presence of amide protons, while those around 3.70 ppm correspond to methoxy protons. The signals for the aromatic protons appear in the range of 6.75–8.20 ppm.

### 3.4. Solubility and Thermal Properties of the PAIs

The solubility of the synthesized *m*-**8** and *p*-**8** series PAIs was evaluated in various organic solvents, including DMSO, DMAc, DMF, NMP, *m*-cresol, and THF, as summarized in Table 1. Most of the *m*-**8** series polymers demonstrated good solubility in polar aprotic solvents such as NMP, DMSO, DMAc, and DMF, but remained insoluble in the less polar solvent THF. This solubility is advantageous for solution processing, particularly in film casting applications. The relatively high solubility of the *m*-**8** series is likely due to the incorporation of *meta*-oriented benzamide linkages and methoxy-substituted TPPA side chains, both of which introduce structural distortion and reduce interchain packing. The propeller-shaped triphenylamine cores further disrupt planarity and enhance the solvation of the polymer chains. In contrast, the *p*-**8** series PAIs exhibited noticeably lower solubility. Several samples were only partially soluble or required heating to dissolve in polar solvents like DMSO and DMF, while most remained insoluble in the less polar THF. The reduced solubility can be attributed to the more linear *para*-oriented benzamide structures, which facilitate tighter chain packing and stronger intermolecular interactions, thereby limiting solvent accessibility and dispersion.

The glass transition temperatures (*T*_g_) of the *m*-**8** and *p*-**8** series PAIs were analyzed using differential scanning calorimetry (DSC), with results summarized in Table 2 and depicted in Figure S10. Prior to measurement, each polymer sample was heated to 350 °C to remove any residual crystallinity and rapidly cooled to ensure a predominantly amorphous matrix. Obvious baseline shifts could be observed in the second DSC heating traces, and the *T*_g_ was defined as the midpoint temperature of the baseline shift. The *m*-**8** series polymers displayed *T*_g_ values in the range of 250 to 277 °C, while the glass transition of the *p*-**8** series PAIs occurred between 252 and 292 °C. The observed trend reflects the influence of molecular geometry on chain mobility. The *meta*-connected benzamide units introduce more torsional flexibility into the backbone, leading to lower thermal transition temperatures. In contrast, the *para*-oriented structures create a more linear and rigid backbone, which restricts segmental motion and results in elevated *T*_g_ values. PAIs *m*-**8c** and *p*-**8c** exhibited the lowest *T*_g_ in their respective series due to the presence of flexible ether linkages from the OPDA component. These PAIs generally exhibited a higher *T*_g_ compared to the corresponding polyamides due to the incorporation of rigid imide rings. For example, the *T*_g_ of PAI *p*-**8d** was recorded at 278 °C, while the *T*_g_ of the structurally related polyamide derived from TPPA-diamine **4** and 4,4′-oxydibenzoic acid was 248 °C [31].

The thermal stability of the PAIs was assessed through thermogravimetric analysis (TGA), with the corresponding curves displayed in Figure S11. All polymers exhibited high thermal resistance, showing negligible weight loss before reaching 400 °C. The temperatures corresponding to 5% and 10% weight loss were consistently above 410 °C and 460 °C, respectively, indicating that these materials can withstand elevated processing and operating conditions. Under nitrogen, the polymers retained over 50% of their weight at 800 °C, which can be attributed to their rigid aromatic backbones and stable molecular structures. Thermal degradation in air began slightly earlier than in nitrogen, as is typical for oxidative environments; however, the materials still demonstrated excellent thermal stability. These findings confirm that both the *m*-**8** and *p*-**8** series PAIs are thermally robust under both inert and oxidative conditions, supporting their potential use in high-temperature applications. Among the tested polymers, the samples prepared from BPDA (*m*-**8b** and *p*-**8b**) exhibited the highest thermal stability, attributed to the chain rigidity conferred by the rigid biphenyl structure. In contrast, the PAIs *m*-**8e** and *p*-**8e**, derived from DSDA, displayed lower decomposition temperatures due to the less thermally stable sulfonyl linkage present in the DSDA component. These results underscore the significant influence of the dianhydride unit structures on the heat resistance of the final polymers.

### 3.5. Electrochemical Properties

To investigate the electrochemical characteristics of the triarylamine-containing model structures and assess how molecular features influence redox behavior, cyclic voltammetry (CV) analyses were conducted on model compounds **M1** and **M2**. These compounds were designed to represent structural units of the target polymers, with variations in functional groups facilitating direct comparison. **M1** contains amide functionalities with N–H moieties, which may slightly donate electrons, while **M2** incorporates only electron-withdrawing imide units. Comparing their oxidation profiles reveals how different linkages affect the redox potential. These low-molecular-weight compounds serve as simplified analogs, allowing for clearer observation of intrinsic redox features without the complexity of lengthy polymer chains. All measurements were performed in anhydrous acetonitrile with 0.1 M Bu_4_NClO_4_ as the supporting electrolyte.

**Figure 6:**
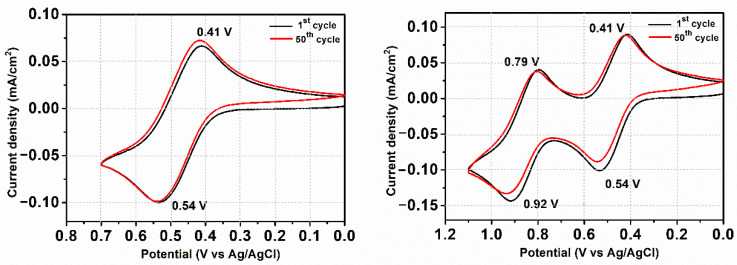
CV diagrams of **M1** on an ITO glass slide in 0.1 M Bu_4_NClO_4_/MeCN at a scan rate of 50 mV/s for 50 cycles.

**Figure 7:**
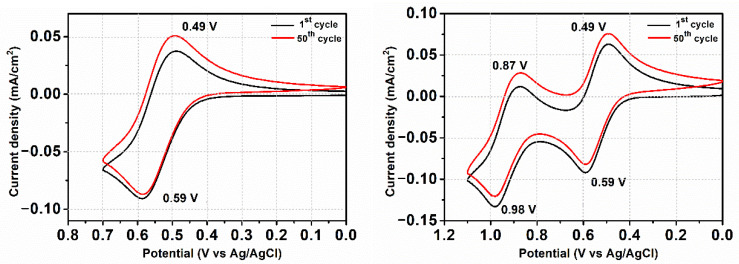
CV diagrams of **M2** on an ITO glass slide in 0.1 M Bu_4_NClO_4_/MeCN at a scan rate of 50 mV/s for 50 cycles.

The CV diagrams for the solution of model compound **M1** across two different potential ranges are presented in Figure 6. In the first CV scan, which swept from 0 to 1.1 V, **M1** exhibited two oxidation waves at 0.54 and 0.92 V. These two redox processes are reversible, as indicated by equal peak currents for the forward and reverse waves. The first electron removal for **M1** is believed to occur at the nitrogen atom in the pendant 4,4′-dimethoxytriphenylamine unit, which is more electron-rich than the nitrogen atom in the other triphenylamine (TPA) unit. The strong electron-donating effect of the methoxy groups lowers the oxidation potential and stabilizes the resulting radical cation. Consequently, nearly no current decrease was observed during the first 50 repetitive CV scans over the potential range of 0 to 0.7 V, indicating highly stable redox behavior for the first redox process. As the potential was swept between 0 and 1.1 V for 50 continuous scans, **M1** maintained high electrochemical activity and reversibility, suggesting that the second redox process was also highly stable. As shown in Figure 7, model compound **M2** exhibited two oxidation waves at 0.59 V and 0.98 V, slightly higher than those of **M1** due to the TPPA unit being directly linked to the electron-withdrawing imide ring. Nonetheless, **M2** also demonstrated good electrochemical stability during both the first and second redox processes, as evidenced by the lack of significant current decline in its CV curve after 50 continuous scans. Thus, the dimethoxy-substituted TPPA is an ideal unit for constructing electroactive polymers for use in optoelectronic devices.

The redox behavior and electrochemical stability of the synthesized PAIs were also investigated using CV, with polymer-coated indium tin oxide (ITO) glass substrates serving as the working electrode, a saturated Ag/AgCl electrode as the reference electrode, and 0.1 M tetrabutylammonium perchlorate (TBAP; Bu_4_NClO_4_) in anhydrous acetonitrile.

(MeCN) as the supporting electrolyte. The electrochemical behaviors of all PAIs are illustrated in Figures S12–S20, and the electrochemical data, along with the UV–vis absorption wavelengths of the PAIs, are summarized in Table 3. All PAIs exhibited two pairs of reversible redox waves, with half-wave potentials recorded in the ranges of 0.46–0.53 V (*E*_1/2_^Ox1^) and 0.84–0.89 V (*E*_1/2_^Ox2^) for the first and second redox processes, respectively. The oxidation potentials of these PAIs are comparable to those reported for the polyamide analogues (*E*_1/2_^Ox1^ = 0.47–0.51 V and *E*_1/2_^Ox2^ = 0.82–0.86 V) [31], as they share the same TPPA segment and linking groups.

**Figure 8:**
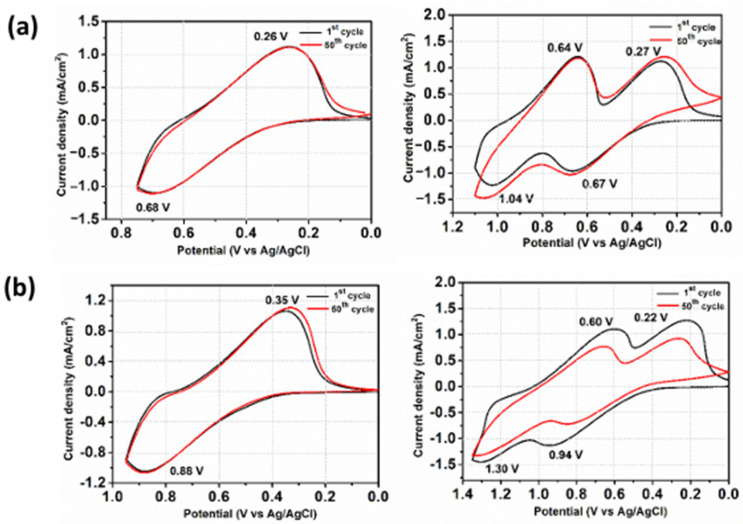
CV diagrams of (**a**) PAI *m*-**8c** and (**b**) PI**-8c′** films on ITO glass slide in 0.1 M Bu_4_NClO_4_/MeCN at a scan rate of 50 mV/s in different potential ranges.

Taking PAI *m*-**8c** as an example, this polymer displayed an oxidation onset potential (*E*_onset_) at 0.33 V, followed by two consecutive oxidation waves at 0.67 V and 1.04 V (Figure 8a). PAI *m*-**8c** exhibited higher oxidation potentials than model compound **M1** (*E*_pa_^Ox1^ = 0.54 V, *E*_pa_^Ox2^ = 0.92 V) because the diffusion rate of ^−^ClO_4_ ion in the polymer film is slower than in solution. The first oxidation reaction is believed to occur on the pendent dimethoxy-TPA unit, followed by the second electron removal from the TPPA radical cation. During the first 50 CV cycles, no significant decline in current was observed for the first redox process, indicating high electrochemical stability. This stability can be partly attributed to the electron-donating methoxy substituents on the redox-active TPPA segment. However, when the potential was swept between 0 and 1.1 V (just over the second oxidation stage) for 50 repetitive scans, a slight decrease in electrochemical reversibility was noticed.

For comparison, the CV diagrams of the reference polymer PI-**8c′** were analyzed and are presented in Figure 8b. This polymer exhibited higher oxidation potentials (*E*_pa_^Ox1^ = 0.88 V, *E*_pa_^Ox2^ = 1.30 V) than the corresponding PAI *m*-**8c** (*E*_pa_^Ox1^ = 0.67 V, *E*_pa_^Ox2^ = 1.04 V) due to the direct connection of the electron-withdrawing imide ring to the TPPA segment. The first oxidized state of PI-**8c′** was stable enough that, after 50 CV cycles, no significant changes were observed in its CV profile for the first redox process. However, when the potential was increased to 1.35 V for the second oxidation, a gradual decline in electroactivity was noted for PI-**8c′**. Upon oxidation, both the PAI and PI films exhibited distinct color changes, transitioning from colorless to green and then to blue. They also demonstrated good electroactivity and strong adhesion to the ITO-coated glass. Both PAIs and the PI maintained stable redox behavior during the first oxidation stage throughout the initial 50 CV cycles. However, upon scanning into the second oxidation range, a slight decrease in electrochemical reversibility was observed. This may be attributed to the formation of dicationic species, which are less stable due to cumulative electron withdrawal from the TPPA segments.

The electronic energy levels of the synthesized polymers were estimated based on their electrochemical and optical data. The highest occupied molecular orbital (HOMO) energy levels were calculated from the first half-wave oxidation potentials (*E*_1/2_^Ox1^), using the Fc/Fc^+^ redox couple (−4.80 eV vs. vacuum) as a reference standard. The measurements indicated that the HOMO levels of the polymers ranged from −4.82 to −4.89 eV. The lowest unoccupied molecular orbital (LUMO) energy levels were estimated by subtracting the optical band gap (*E*_g_^opt^), obtained from the UV–Vis absorption edge, from the corresponding HOMO values. The calculated LUMO levels ranged from −1.83 to −2.01 eV, as summarized in Table 3.

### 3.6. Spectroelectrochemical and Electrochromic Properties

The spectroelectrochemical behavior of the polymer films was investigated by recording UV–Vis absorption spectra at various applied potentials. Polymer films were cast onto ITO-coated glass substrates, which were then placed in a cuvette filled with 0.1 M Bu_4_NClO_4_ in acetonitrile. A standard three-electrode configuration, identical to that used in the CV setup, was employed. As increasing voltages were applied, the films exhibited visible color changes, accompanied by distinct shifts in their absorption spectra, thereby confirming their electrochromic properties. The UV-vis-NIR absorption profile of pure ITO glass is shown in Figure S21. The substrate is essentially transparent within the wavelength range of 300–1000 nm.

**Figure 9:**
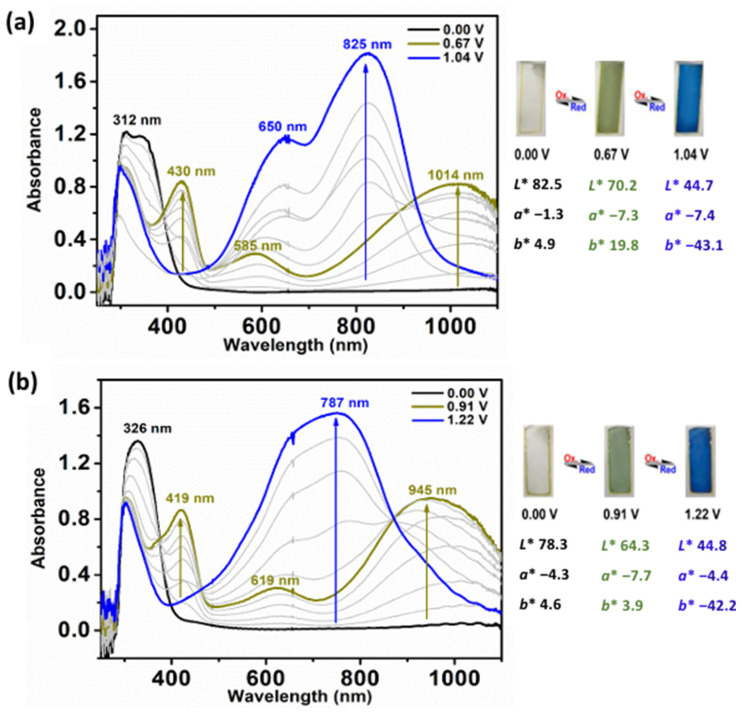
Spectroelectrograms and color changes of (**a**) PAI ***m*-8c** and (**b**) PI-**8c′** on an ITO glass slide in 0.1 M Bu_4_NClO_4_/MeCN at various applied voltages.

Typical spectral and color changes of PAI *m*-**8c** under varying applied potentials are illustrated in Figure 9a. In its neutral state, the film displayed a strong absorption band at 312 nm, corresponding to π − π* transitions in the conjugated units. The film exhibited minimal absorption in the visible and near-infrared (NIR) regions, appearing nearly colorless. Upon oxidation at 0.67 V, new peaks emerged at 430, 585, and 1014 nm, resulting in a green coloration (L*: 70.2; a*: −7.3; b*: 19.8). The absorptions at 430 and 585 nm are attributed to the generation of a nitrogen-centered radical cation, while the broad NIR absorption at 1014 nm arises from intervalence charge transfer (IVCT) between partially oxidized nitrogen centers. As the voltage was further increased to 1.04 V, the NIR peak gradually diminished, and new bands appeared at 650 and 825 nm, indicating the formation of dications in the TPPA unit. This led to a color change from green to blue (L*: 44.7; a*: −7.4; b*: −43.1), consistent with typical two-stage electrochromic transitions of the TPPA-based polymers [31,32,33,34,35]. The other PAIs exhibited very similar spectral and color changes to those of *m*-**8c** (Figures S22 and S23), indicating that despite differences in the dianhydride components or *p*-/*m*-connected benzamide spacers, the optical behavior of TPPA during oxidation remains consistent.

The absorption profile and color changes of the reference PI**-8c′** film under various applied voltages are illustrated in Figure 9b. In its neutral state, the PI-**8c′** film exhibited an absorption peak at 326 nm. As the potential was increased to 0.91 V, new bands appeared at 419, 619, and 945 nm, accompanied by a visible green hue (L*: 64.3; a*: −7.7; b*: 3.9). At a higher voltage of 1.22 V, a strong and broad absorption centered at 787 nm developed, and the film transitioned to blue (L*: 44.8; a*: −4.4; b*: −42.2). PI-**8c′** exhibited slightly blue-shifted and broader absorption bands compared to the structurally related PAI *m*-**8c**. This difference is likely due to the absence of benzamide linkages in PI-**8c′**. Unlike imide-only backbones, the presence of a benzamide spacer between the TPPA unit and the imide ring provides an extended resonance domain for the radical cation and dicationic species, enhancing intramolecular charge transfer and leading to red-shifted NIR absorption.

### 3.7. Electrochromic Switching Stability

The electrochromic switching performance of the polymer films was evaluated by monitoring the time-dependent transmittance (%T) at their characteristic absorption maxima (λ_max_) during potential step tests. A square-wave voltage was applied to the drop-cast polymer film on an ITO-coated glass slide immersed in a 0.1 M Bu_4_NClO_4_/acetonitrile solution, and the resulting changes in transmittance were measured using a UV–Vis spectrophotometer. The polymer films were coated onto ITO glass substrates with an active area of approximately 1.76 cm^2^. The switching time was defined as the duration required for the transmittance to reach 90% of its full change between the colored and bleached states, as variations beyond this point are generally imperceptible to the human eye.

**Figure 10:**
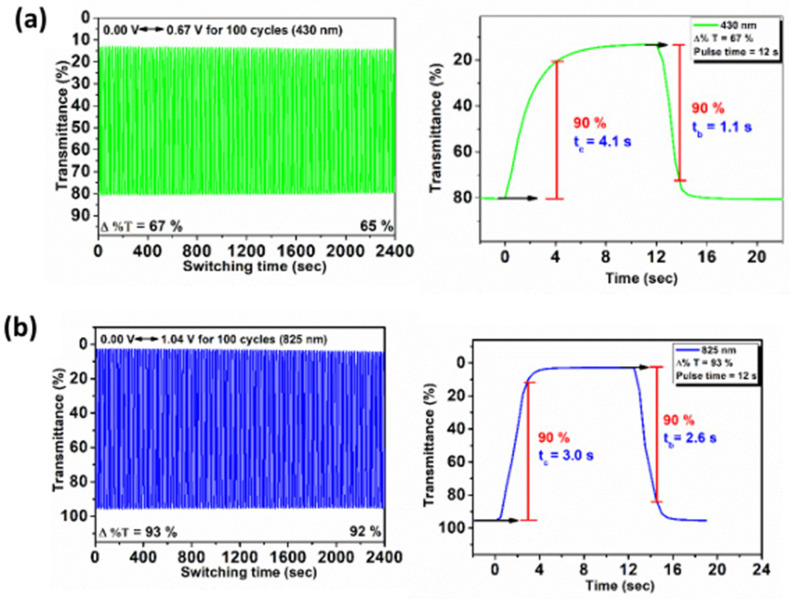
Electrochromic switching responses of the cast film of PAI ***m*-8c** on the ITO glass slide (coated area ~ 0.8 × 2.2 cm^2^) in 0.1 M Bu_4_NClO_4_/MeCN by applying a square-wave potential step between (**a**) 0.00 V and 0.67 V, monitored at λ_max_ = 430 nm, and (**b**) 0.00 V and 1.04 V, monitored at λ_max_ = 825 nm.

For the *m*-**8c** film, switching between the neutral and first oxidized forms was achieved by applying a square-wave potential between 0 and 0.67 V, with a pulse duration of 12 s. The transmittance variation was monitored at 430 nm, where the film exhibited a coloring time of 4.1 s and a bleaching time of 1.1 s. The initial optical contrast (Δ%T) was 67%, remaining nearly unchanged at 65% after 100 switching cycles (Figure 10a), indicating high electrochromic switching stability. The electrochromic film maintained a high contrast of 60% over 1000 switching cycles. As shown in Figure 10b, applying a voltage of up to 1.04 V induced a transition to the second oxidized state. The film exhibited broad absorption at 825 nm, corresponding to a blue coloration, with Δ%T (also known as optical contrast) reaching 93%. After 100 cycles, the optical contrast slightly decreased to 92%, suggesting reliable switching endurance. Even after 1000 switching cycles, this polymer maintained a rapid electrochromic response and a high optical contrast of 85%. Overall, the PAI film demonstrated comparable electrochromic switching stability and optical contrast to its polyamide counterpart reported in the literature [31].

Other PAI films demonstrated Δ%T values ranging from 36% to 67% at 429–430 nm during green-state switching, with most maintaining stable performance during the first 100 cycles (Figures S24–S31). The coloration and bleaching times ranged from 2.6 s to 4.1 s and from 1.1 s to 4.7 s, respectively. For the second-stage oxidation (0–1.04 V), the Δ%T values at 820–832 nm ranged from 75% to 99%. Most films retained high optical contrast after repeated switching, except for some samples derived from DSDA and 6FDA such as *m*-**8d**, *p*-**8d**, and *p*-**8e** (see Figures S26, S30 and S31), which exhibited a slight drop in performance, likely due to partial delamination or surface dissolution attributed to their higher solubility in the electrolyte solution. In general, most of the PAIs exhibited high electrochromic stability in the first 100 switching cycles. The key parameters of electrochromic properties for all the polymer films during electrochemical redox processes are summarized in Table 4.

**Figure 11:**
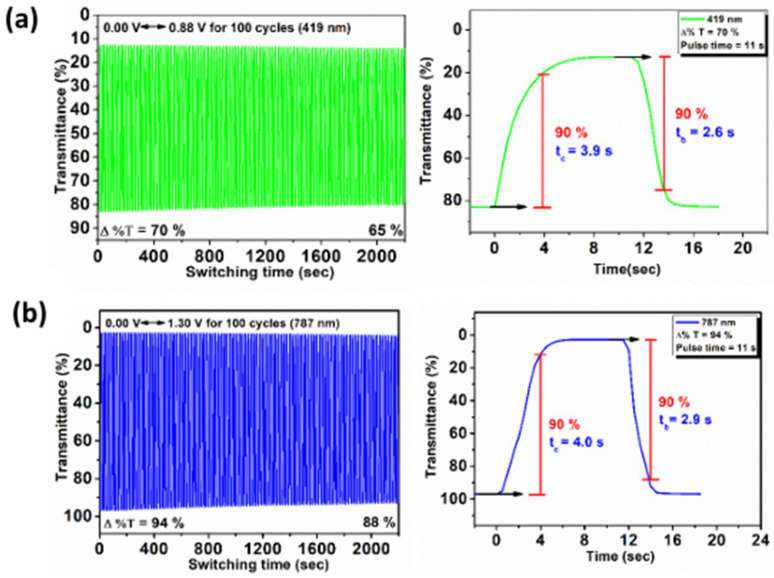
Electrochromic switching responses of the cast film of PI **8c′** on the ITO glass slide (coated area ~ 0.8 × 2.2 cm^2^) in 0.1 M Bu_4_NClO_4_/MeCN by applying a square-wave potential step between (**a**) 0.00 V and 0.88 V, monitored at λ_max_ = 419 nm, and (**b**) 0.00 V and 1.30 V, monitored at λ_max_ = 787 nm.

The electrochromic switching of PI-**8c′** was also examined. At 0.88 V, the film exhibited green coloration at 419 nm with an optical contrast of 70%, which slightly decreased to 65% after 100 cycles. The switching times were 3.9 s for coloration and 2.6 s for bleaching (Figure 11). Upon increasing the voltage to 1.30 V, a blue absorption band emerged at 787 nm. The initial contrast was 94%, but it dropped to 88% after 100 cycles, indicating a more pronounced loss of optical contrast. The blue coloration and bleaching times were 4.0 s and 2.9 s, respectively. Compared to the PAI films, PI-**8c′** exhibited a more significant decline in optical contrast across both oxidation stages during repeated switching. This overall reduction in electrochromic stability may be attributed to the absence of amide functionalities in the PI backbone, which contribute to charge stabilization through hydrogen bonding and conformational rigidity. Without these stabilizing interactions, the oxidized species are more susceptible to degradation or structural reorganization, leading to diminished reversibility and optical durability.

## 4. Conclusions

Two novel TPPA-based isomeric diamide–diamine monomers, *N*,*N*-bis(4-(3-aminobenzamido)phenyl)-*N*’,*N*’-bis(4-methoxyphenyl)-1,4-phenylenediamine (*m*-**6**) and *N*,*N*-bis(4-(4-aminobenzamido)phenyl)-*N*’,*N*’-bis(4-methoxyphenyl)-1,4-phenylenediamine (*p*-**6**), were successfully synthesized. These monomers were subsequently polymerized with various aromatic dianhydrides to produce two isomeric series of electroactive aromatic poly(amide-imide)s (PAIs), featuring *m*- or *p*-linked benzamide spacers between the TPPA segment and the imide ring. The PAIs demonstrated reasonable thermal stability, with glass transition temperatures (*T*_g_) ranging from 250 to 292 °C and no significant degradation observed before 400 °C. Almost all PAIs could be solution-cast into homogeneous and adherent thin films on ITO glass substrates, serving as electrodes for electrochemical and electrochromic tests. Cyclic voltammetry (CV) measurements showed that all PAIs exhibited two reversible redox couples at *E*_1/2_^Ox1^ = 0.46–0.53 V and *E*_1/2_^Ox2^ = 0.84–0.89 V (vs. Ag/AgCl) in an electrolyte-containing acetonitrile solution, accompanied by strong color transitions from neutral colorless to green and blue in the oxidized states. During 50 repetitive CV cycles, all polymers displayed high redox reversibility and stability, with minimal decay and almost no deviation in the maximum current peaks. The PAIs showcased outstanding electrochromic performance, including high optical contrast (up to 95%), rapid response times (below 4.6 s for coloring and 5.7 s for bleaching), high coloration efficiency (up to 240 cm^2^/C), and low decay in optical contrast (less than 5% after 100 switching cycles for most PAIs). Compared to the corresponding polyimide without the benzamide spacer, the PAIs exhibited higher solubility, lower oxidation potentials, and enhanced electrochemical and electrochromic stability, particularly during the second oxidation stage. These positive results suggest that the PAI materials developed in this work hold great potential for applications in electrochromic and other optoelectronic devices.

## Figures and Tables

**Table 1:** Inherent viscosity and solubility behaviors of polymers.

Polymer Code	η_inh_ ^a^ (dL/g)	Solvents ^b,c^					
		NMP	DMAc	DMF	DMSO	*m*-Cresol	THF
***m*** **-8a**	0.35	++	++	+h	++	+−	−
***m*** **-8b**	0.44	+h	+h	+−	+h	+−	−
***m*** **-8c**	0.31	++	++	++	++	+h	−
***m*** **-8d**	0.36	++	++	++	++	+h	+−
***m*** **-8e**	0.85	++	++	+h	++	+h	−
***p*** **-8b**	0.46	+h	+h	+−	+h	+−	−
***p*** **-8c**	0.33	++	++	+h	++	+−	+−
***p*** **-8d**	0.42	++	++	++	++	+h	+−
***p*** **-8e**	0.71	+h	+h	+−	+h	+−	−
**PI-8c′**	0.42	++	++	+−	+−	−	−

^a^ Inherent viscosity measured at a concentration of 0.5 g/dL in DMAc at 30 °C. ^b^ The qualitative solubility was tested with 10 mg of a sample in 1 mL of stirred solvent. ++: soluble at room temperature; +h: soluble on heating +−: partially soluble; −: insoluble even on heating. ^c^ Solvents: NMP: *N*-methyl-2-pyrrolidone; DMAc: *N*,*N*-dimethylacetamide; DMF: *N*,*N*-dimethylformamide; DMSO: dimethyl sulfoxide; THF: tetrahydrofuran.

**Table 2:** Thermal properties of polymers.

Polymer Code	*T*_g_ (°C) ^a^	*T*_d_ at 5% Weight Loss (°C) ^b^		*T*_d_ at 10% Weight Loss (°C) ^b^		Char Yield (wt %) ^c^
		N_2_	Air	N_2_	Air	
***m*** **-8a**	268	445	417	469	469	58
***m*** **-8b**	277	464	458	489	494	55
***m*** **-8c**	250	457	457	483	499	48
***m*** **-8d**	267	468	428	493	485	54
***m*** **-8e**	265	435	421	461	460	52
***p*** **-8b**	292	468	465	494	501	59
***p*** **-8c**	252	455	461	477	489	56
***p*** **-8d**	278	470	465	498	501	54
***p*** **-8e**	275	439	437	465	472	55
**PI-8c′**	244	457	473	481	525	48

^a^ Midpoint temperature of the baseline shift on the second DSC heating trace (rate = 20 °C/min) after quenching from 350 °C to 50 °C in nitrogen. ^b^ Decomposition temperature at which a 5% or 10% weight loss was recorded by TGA at a heating rate of 20 °C/min and a gas flow rate of 20 cm^3^/min. ^c^ Carbonized residue percentages at 800 °C at a scan rate 20 °C/min in nitrogen.

**Table 3:** Optical and electrochemical properties of polymers.

Polymer Code	Thin film Absorption Wavelength (nm)		Oxidation Potential (V) ^a^			*E*_g_^opt^ (eV) ^b^	HOMO (eV) ^c^	LUMO (eV) ^d^
	λ_max_	λ_onset_	*E*_onset_	*E*_1/2_^Ox1^	*E*_1/2_^Ox2^			
***m*** **-8a**	314	424	0.34	0.47	0.87	2.92	−4.83	−1.91
***m*** **-8b**	338	422	0.34	0.47	0.86	2.94	−4.83	−1.89
***m*** **-8c**	312	422	0.33	0.47	0.84	2.94	−4.83	−1.89
***m*** **-8d**	304	419	0.34	0.47	0.85	2.96	−4.83	−1.87
***m*** **-8e**	308	422	0.34	0.46	0.85	2.94	−4.82	−1.88
***p*** **-8b**	311	422	0.35	0.50	0.88	2.94	−4.86	−1.92
***p*** **-8c**	308	425	0.42	0.53	0.87	2.92	−4.89	−1.97
***p*** **-8d**	304	424	0.34	0.49	0.88	2.92	−4.85	−1.93
***p*** **-8e**	304	420	0.37	0.52	0.89	2.95	−4.88	−1.93
PI**-8c′**	326	402	0.46	0.62	0.95	3.09	−4.98	−1.89

^a^ vs. Ag/AgCl in CH_3_CN. *E*_1/2_ = average potential of redox couple peaks. ^b^ Bandgap calculated from the absorption edge of the polymer film: Energy gap = 1240/λ_onset_. ^c^ The HOMO energy levels were calculated from *E*_1/2_^Ox1^ values of CV curves and were referenced to ferrocene (4.8 eV relative to the vacuum energy level). *E*_1/2_ = 0.44 V. ^d^ *E*_HOMO_ = −(*E*_1/2_^Ox1^ + 4.8 − 0.44) (eV); *E*_LUMO_ = *E*_HOMO_ + *E*_g_^opt^.

**Table 4:** Electrochromic properties of polymers.

Polymer	λ_max_ ^a^ (nm)	1st Cycle Δ%T	100th Cycle Δ%T	Response Time ^b^		ΔOD ^c^	Q_d_ ^d^ (mC/cm^2^)	CE ^e^ (cm^2^/C)
				t_c_ (s)	t_b_ (s)			
***m*** **-8a**	430	43	42	2.7	4.5	1.39	7.88	176
	825	82	81	4.0	5.7	2.31	10.78	214
***m*** **-8b**	429	61	60	3.5	3.1	0.86	5.03	171
	824	99	96	3.4	4.0	2.57	10.70	240
***m*** **-8c**	430	67	65	4.1	1.1	0.78	3.40	229
	825	93	92	3.0	2.6	1.57	6.52	241
***m*** **-8d**	429	44	43	2.6	1.5	0.31	3.42	91
	820	93	85	4.1	3.6	1.33	9.09	146
***m*** **-8e**	429	47	44	3.7	3.0	0.69	4.26	162
	824	98	97	3.0	3.2	1.95	9.74	200
***p*** **-8b**	430	49	48	3.0	2.3	0.73	4.52	162
	830	96	91	3.7	2.8	1.55	10.4	149
***p*** **-8c**	429	48	47	4.6	4.7	0.38	1.87	203
	832	77	76	3.2	2.6	0.83	4.47	186
***p*** **-8d**	430	46	44	3.6	2.0	0.34	3.64	94
	828	75	59	3.8	2.1	0.64	9.88	65
***p*** **-8e**	429	36	35	3.8	1.6	0.24	2.57	94
	831	75	60	3.6	2.7	0.62	9.22	67
**PI-8c′**	419	70	65	3.9	2.6	0.81	4.46	182
	787	94	88	4.0	2.9	1.62	8.38	193

^a^ Wavelength of absorption maximum. ^b^ Time for 90% of the full-transmittance change. ^c^ Optical Density (ΔOD) = log[T_bleached_/T_colored_], where T_colored_ and T_bleached_ are the maximum transmittance in the oxidized and neutral states, respectively. ^d^ Q_d_ is ejected charge, determined from the in situ experiments. ^e^ Coloration efficiency (CE) = ΔOD/Q_d_.

## Data Availability

The original contributions presented in this study are included in this article. Further inquiries can be directed to the corresponding author.

## Supplementary Materials

The following supporting information can be downloaded at: https://www.mdpi.com/article/10.3390/polym18010139/s1, FTIR, ^1^H NMR, and ^13^C NMR spectra of the prepared compounds and polymers, DSC and TGA curves, CV and spectroelectrochemical diagrams, and electrochromic switching responses of the polymer films. Figure S1: IR spectra of diamine monomer **4** and its precursor compounds. Figure S2: IR spectra of diamide-dinitro compounds *m*-**5** and *p*-**5** and diamide-diamine monomers *m*-**6** and *p*-**6**. Figure S3: ^1^H NMR and H-H COSY NMR spectra of diamide-dinitro compound ***m*-5** in DMSO-*d*_6_. Figure S4: ^1^H NMR and H-H COSY NMR spectra of diamide-dinitro compound ***p*-5** in DMSO-*d*_6_. Figure S5: ^1^H NMR and H-H COSY NMR spectra of diamide-diamine monomer ***m*-6** in DMSO-*d*_6_. Figure S6: ^1^H NMR and H-H COSY NMR spectra of diamide-diamine monomer ***p*-6** in DMSO-*d*_6_. Figure S7: IR spectra of model compounds **M1** and **M2**. Figure S8: IR spectra of poly(amide-imide)s ***m*-8d** and ***p*-8b**. Figure S9: ^1^H NMR spectra of PAIs (a) ***m*-8d** and (b) ***p*-8d** in DMSO-*d*_6_. Table S1: Inherent viscosity and GPC data of PAIs. Figure S10: (a) DSC curves of PAIs (a) ***m*-8a** to ***m*-8e** and (b) ***p*-8b** to ***p*-8e** with a heating rate of 20 °C/min in nitrogen. Figure S11. TGA thermograms of PAIs (a) ***m*-8a** to ***m*-8e** in nitrogen, (b) ***m*-8a** to ***m*-8e** in air, (c) ***p*-8b** to ***p*-8e** in nitrogen, and (d) ***p*-8b** to ***p*-8e** in air, with a heating rate of 20 °C/min. Figure S12: CV diagrams of PAI *m*-**8a** film on ITO-glass slide in 0.1 M Bu_4_NClO_4_/MeCN at a scan rate of 50 mV/s: (a) first scan in the range of 0–0.8 V, (b) comparison of the first and 50^th^ cycles in the range of 0–0.8 V, and (c) comparison of the first and 50^th^ cycles in the range of 0–1.2 V. Figure S13: CV diagrams of PAI *m*-**8b** film on ITO-glass slide in 0.1 M Bu_4_NClO_4_/MeCN at a scan rate of 50 mV/s: (a) first scan in the range of 0–0.8 V, (b) comparison of the first and 50^th^ cycles in the range of 0–0.8 V, and (c) comparison of the first and 50^th^ cycles in the range of 0–1.15 V. Figure S14: CV diagrams of PAI *m*-**8c** film on ITO-glass slide in 0.1 M Bu_4_NClO_4_/MeCN at a scan rate of 50 mV/s: (a) first scan in the range of 0–0.75 V, (b) comparison of the first and 50^th^ cycles in the range of 0–0.75 V, and (c) comparison of the first and 50^th^ cycles in the range of 0–1.1 V. Figure S15: CV diagrams of PAI *m*-**8d** film on ITO-glass slide in 0.1 M Bu_4_NClO_4_/MeCN at a scan rate of 50 mV/s: (a) first scan in the range of 0–0.8 V, (b) comparison of the first and 50^th^ cycles in the range of 0–0.8 V, and (c) comparison of the first and 50^th^ cycles in the range of 0–1.15 V. Figure S16: CV diagrams of PAI m-**8e** film on ITO-glass slide in 0.1 M Bu_4_NClO_4_/MeCN at a scan rate of 50 mV/s: (a) first scan in the range of 0–0.8 V, (b) comparison of the first and 50^th^ cycles in the range of 0–0.8 V, and (c) comparison of the first and 50^th^ cycles in the range of 0–1.15 V. Figure S17: CV diagrams of PAI *p*-**8b** film on ITO-glass slide in 0.1 M Bu_4_NClO_4_/MeCN at a scan rate of 50 mV/s: (a) first scan in the range of 0–0.8 V, (b) comparison of the first and 50^th^ cycles in the range of 0–0.8 V, and (c) comparison of the first and 50^th^ cycles in the range of 0–1.15 V. Figure S18: CV diagrams of PAI *p*-**8c** film on ITO-glass slide in 0.1 M Bu_4_NClO_4_/MeCN at a scan rate of 50 mV/s: (a) first scan in the range of 0–0.85 V, (b) comparison of the first and 50^th^ cycles in the range of 0–0.85 V, and (c) comparison of the first and 50^th^ cycles in the range of 0–1.15 V. Figure S19: CV diagrams of PAI *p*-**8d** film on ITO-glass slide in 0.1 M Bu_4_NClO_4_/MeCN at a scan rate of 50 mV/s: (a) first scan in the range of 0–0.8 V, (b) comparison of the first and 50^th^ cycles in the range of 0–0.8 V, and (c) comparison of the first and 50^th^ cycles in the range of 0–1.15 V. Figure S20: CV diagrams of PAI *p*-**8e** film on ITO-glass slide in 0.1 M Bu_4_NClO_4_/MeCN at a scan rate of 50 mV/s: (a) first scan in the range of 0–0.75 V, (b) comparison of the first and 50^th^ cycles in the range of 0–0.75 V, and (c) comparison of the first and 50th cycles in the range of 0–1.15 V. Figure S21: The UV-vis-NIR absorption profile of pure ITO-glass. Figure S22: Spectroelectrograms and color changes of the cast films of PAIs (a) ***m*-8a**, (b) ***m*-8b**, (c) ***m*-8d**, and (d) ***m*-8e** on an ITO-glass slide in 0.1 M Bu_4_NClO_4_/MeCN at various applied voltages. Figure S23: Spectroelectrograms and color changes of the cast films of PAIs (a) ***p*-8b**, (b) ***p*-8c**, (c) ***p*-8d**, and (d) ***p*-8e** on an ITO-glass slide in 0.1 M Bu_4_NClO_4_/MeCN at various applied voltages. Figure S24: Electrochromic switching responses of the cast film of PAI *m*-**8a** on the ITO-glass slide (coated area ~ 0.8 × 2.2 cm^2^) in 0.1 M Bu_4_NClO_4_ (TBAP)/MeCN by applying a square-wave potential step between (a) 0.00 V and 0.76 V, monitored at λ_max_ = 430 nm and (b) 0.00 V and 1.16 V monitored at λ_max_ = 825 nm. Figure S25: Electrochromic switching responses of the cast film of PAI *m*-**8b** on the ITO-glass slide (coated area ~ 0.8 × 2.2 cm^2^) in 0.1 M TBAP/MeCN by applying a square-wave potential step between (a) 0.00 V and 0.73 V, monitored at λ_max_ = 429 nm and (b) 0.00 V and 1.06 V monitored at λ_max_ = 824 nm. Figure S26: Electrochromic switching responses of the cast film of PAI *m*-**8d** on the ITO-glass slide (coated area ~ 0.8 × 2.2 cm^2^) in 0.1 M TBAP/MeCN by applying a square-wave potential step between (a) 0.00 V and 0.67 V, monitored at λ_max_ = 429 nm and (b) 0.00 V and 1.10 V monitored at λ_max_ = 820 nm. Figure S27: Electrochromic switching responses of the cast film of PAI *m*-**8e** on the ITO-glass slide (coated area ~ 0.8 × 2.2 cm^2^) in 0.1 M TBAP/MeCN by applying a square-wave potential step between (a) 0.00 V and 0.73 V, monitored at λ_max_ = 429 nm and (b) 0.00 V and 1.11 V monitored at λ_max_ = 824 nm. Figure S28: Electrochromic switching responses of the cast film of PAI *p*-**8b** on the ITO-glass slide (coated area ~ 0.8 × 2.2 cm^2^) in 0.1 M TBAP/MeCN by applying a square-wave potential step between (a) 0.00 V and 0.73 V, monitored at λ_max_ = 430 nm and (b) 0.00 V and 1.16 V monitored at λ_max_ = 830 nm. Figure S29: Electrochromic switching responses of the cast film of PAI *p*-**8c** on the ITO-glass slide (coated area ~ 0.8 × 2.2 cm^2^) in 0.1 M TBAP/MeCN by applying a square-wave potential step between (a) 0.00 V and 0.81 V, monitored at λ_max_ = 429 nm and (b) 0.00 V and 1.09 V monitored at λ_max_ = 832 nm. Figure S30: Electrochromic switching responses of the cast film of PAI *p*-**8d** on the ITO-glass slide (coated area ~ 0.8 × 2.2 cm^2^) in 0.1 M TBAP/MeCN by applying a square-wave potential step between (a) 0.00 V and 0.73 V, monitored at λ_max_ = 430 nm and (b) 0.00 V and 1.10 V monitored at λ_max_ = 828 nm. Figure S31: Electrochromic switching responses of the cast film of PAI *p*-**8e** on the ITO-glass slide (coated area ~ 0.8 × 2.2 cm^2^) in 0.1 M TBAP/MeCN by applying a square-wave potential step between (a) 0.00 V and 0.69 V, monitored at λ_max_ = 429 nm and (b) 0.00 V and 1.10 V monitored at λ_max_ = 831 nm
