# Supplementary material for: Dual-Band Electrochromic Poly(Amide-Imide)s with Redox-Stable N,N,N’,N’-Tetraphenyl-1,4-Phenylenediamine Segments
Source: Polymers (Basel). 2026 Jan 3;18(1):139. doi: 10.3390/polym18010139 (PMC12787878; doi:10.3390/polym18010139)
Supplement: Supplementary file 1 [file polymers-18-00139-s001.zip › polymers-4048314-supplementary.pdf]

Supplementary Materials

Dual-Band Electrochromic Poly(amide-imide)s with Redox-Stable *N,N,N′,N′*-Tetraphenyl-1,4-phenylenediamine Segments

Bo-Wei Huang and Sheng-Huei Hsiao*

Department of Chemical Engineering and Biotechnology, National Taipei University of Technology, Taipei 10451, Taiwan; s0979121791@gmail.com
*Correspondence: shhsiao@ntut.edu.tw

Table of Contents

Figure S1. IR spectra of diamine monomer **4** and its precursor compounds.................4
Figure S2. IR spectra of diamide-dinitro compounds ***m*-5** and ***p*-5** and diamide-diamine monomers ***m*-6** and ***p*-6**...5
Figure S3. ¹H NMR and H-H COSY NMR spectra of diamide-dinitro compound ***m*-5** in DMSO-d_6...6
Figure S4. ¹H NMR and H-H COSY NMR spectra of diamide-dinitro compound ***p*-5** in DMSO-d_6...7
Figure S5. ¹H NMR and H-H COSY NMR spectra of diamide-diamine monomer ***m*-6** in DMSO-d_6...8
Figure S6.¹H NMR and H-H COSY NMR spectra of diamide-diamine monomer ***p*-6** in DMSO-d_6...9
Figure S7. IR spectra of model compounds **M1** and **M2**...10
Figure S8. IR spectra of poly(amide-imide)s ***m*-8d** and ***p*-8b**.......................................11
Figure S9. ¹H NMR spectra of PAIs (a) ***m*-8d** and (b) ***p*-8d** in DMSO-d_6.........................12
Table S1. Inherent viscosity and GPC data of PAIs..13
Figure S10. (a) DSC curves of PAIs (a) ***m*-8a** to ***m*-8e** and (b) ***p*-8b** to ***p*-8e** with a heating rate of 20 °C/min in nitrogen..14
Figure S11. TGA thermograms of PAIs (a) ***m*-8a** to ***m*-8e** in nitrogen, (b) ***m*-8a** to ***m*-8e** in air, (c) ***p*-8b** to ***p*-8e** in nitrogen, and (d) ***p*-8b** to ***p*-8e** in air, with a heating rate of 20 °C/min...15
Figure S12. CV diagrams of PAI *m*-**8a** film on ITO-glass slide in 0.1 M Bu_4NClO_4/MeCN at a scan rate of 50 mV/s: (a) first scan in the range of 0–0.8 V, (b) comparison of the first and 50th cycles in the range of 0–0.8 V, and (c) comparison of the first and 50th cycles in the range of 0–1.2 V...16

Figure S13. CV diagrams of PAI *m*-**8b** film on ITO-glass slide in 0.1 M Bu_4NClO_4/MeCN at a scan rate of 50 mV/s: (a) first scan in the range of 0–0.8 V, (b) comparison of the first and 50th cycles in the range of 0–0.8 V, and (c) comparison of the first and 50th cycles in the range of 0–1.15 V..**17**
Figure S14. CV diagrams of PAI *m*-**8c** film on ITO-glass slide in 0.1 M Bu_4NClO_4/MeCN at a scan rate of 50 mV/s: (a) first scan in the range of 0–0.75 V, (b) comparison of the first and 50th cycles in the range of 0–0.75 V, and (c) comparison of the first and 50th cycles in the range of 0–1.1 V..**18**
Figure S15. CV diagrams of PAI *m*-**8d** film on ITO-glass slide in 0.1 M Bu_4NClO_4/MeCN at a scan rate of 50 mV/s: (a) first scan in the range of 0–0.8 V, (b) comparison of the first and 50th cycles in the range of 0–0.8 V, and (c) comparison of the first and 50th cycles in the range of 0–1.15 V..**19**
Figure S16. CV diagrams of PAI *m*-**8e** film on ITO-glass slide in 0.1 M Bu_4NClO_4/MeCN at a scan rate of 50 mV/s: (a) first scan in the range of 0–0.8 V, (b) comparison of the first and 50th cycles in the range of 0–0.8 V, and (c) comparison of the first and 50th cycles in the range of 0–1.15 V..**20**
Figure S17. CV diagrams of PAI *p*-**8b** film on ITO-glass slide in 0.1 M Bu_4NClO_4/MeCN at a scan rate of 50 mV/s: (a) first scan in the range of 0–0.8 V, (b) comparison of the first and 50th cycles in the range of 0–0.8 V, and (c) comparison of the first and 50th cycles in the range of 0–1.15 V..**21**
Figure S18. CV diagrams of PAI *p*-**8c** film on ITO-glass slide in 0.1 M Bu_4NClO_4/MeCN at a scan rate of 50 mV/s: (a) first scan in the range of 0–0.85 V, (b) comparison of the first and 50th cycles in the range of 0–0.85 V, and (c) comparison of the first and 50th cycles in the range of 0–1.15 V..**22**
Figure S19. CV diagrams of PAI *p*-**8d** film on ITO-glass slide in 0.1 M Bu_4NClO_4/MeCN at a scan rate of 50 mV/s: (a) first scan in the range of 0–0.8 V, (b) comparison of the first and 50th cycles in the range of 0–0.8 V, and (c) comparison of the first and 50th cycles in the range of 0–1.15 V..**23**
Figure S20. CV diagrams of PAI *p*-**8e** film on ITO-glass slide in 0.1 M Bu_4NClO_4/MeCN at a scan rate of 50 mV/s: (a) first scan in the range of 0–0.75 V, (b) comparison of the first and 50th cycles in the range of 0–0.75 V, and (c) comparison of the first and 50th cycles in the range of 0–1.15 V..**24**
Figure S21. The UV-vis-NIR absorption profile of pure ITO-glass.................................**25**
Figure S22. Spectroelectrograms and color changes of the cast films of PAIs (a) ***m*-8a**, (b) ***m*-8b**, (c) ***m*-8d**, and (d) ***m*-8e** on an ITO-glass slide in 0.1 M Bu_4NClO_4/MeCN at various applied voltages..**26**
Figure S23. Spectroelectrograms and color changes of the cast films of PAIs (a) ***p*-8b**,

(b) ***p*-8c**, (c) ***p*-8d**, and (d) ***p*-8e** on an ITO-glass slide in 0.1 M Bu_4NClO_4/MeCN at various applied voltages..**27**
Figure S24. Electrochromic switching responses of the cast film of PAI *m*-**8a** on the ITO-glass slide (coated area ~ 0.8 × 2.2 cm^2) in 0.1 M Bu_4NClO_4 (TBAP)/MeCN by applying a square-wave potential step between (a) 0.00 V and 0.76 V, monitored at λ_{max} = 430 nm and (b) 0.00 V and 1.16 V monitored at λ_{max} = 825 nm........................**28**
Figure S25. Electrochromic switching responses of the cast film of PAI *m*-**8b** on the ITO-glass slide (coated area ~ 0.8 × 2.2 cm^2) in 0.1 M TBAP/MeCN by applying a square-wave potential step between (a) 0.00 V and 0.73 V, monitored at λ_{max} = 429 nm and (b) 0.00 V and 1.06 V monitored at λ_{max} = 824 nm..**28**
Figure S26. Electrochromic switching responses of the cast film of PAI *m*-**8d** on the ITO-glass slide (coated area ~ 0.8 × 2.2 cm^2) in 0.1 M TBAP/MeCN by applying a square-wave potential step between (a) 0.00 V and 0.67 V, monitored at λ_{max} = 429 nm and (b) 0.00 V and 1.10 V monitored at λ_{max} = 820 nm..**29**
Figure S27. Electrochromic switching responses of the cast film of PAI *m*-**8e** on the ITO-glass slide (coated area ~ 0.8 × 2.2 cm^2) in 0.1 M TBAP/MeCN by applying a square-wave potential step between (a) 0.00 V and 0.73 V, monitored at λ_{max} = 429 nm and (b) 0.00 V and 1.11 V monitored at λ_{max} = 824 nm..**29**
Figure S28. Electrochromic switching responses of the cast film of PAI *p*-**8b** on the ITO-glass slide (coated area ~ 0.8 × 2.2 cm^2) in 0.1 M TBAP/MeCN by applying a square-wave potential step between (a) 0.00 V and 0.73 V, monitored at λ_{max} = 430 nm and (b) 0.00 V and 1.16 V monitored at λ_{max} = 830 nm..**30**
Figure S29. Electrochromic switching responses of the cast film of PAI *p*-**8c** on the ITO-glass slide (coated area ~ 0.8 × 2.2 cm^2) in 0.1 M TBAP/MeCN by applying a square-wave potential step between (a) 0.00 V and 0.81 V, monitored at λ_{max} = 429 nm and (b) 0.00 V and 1.09 V monitored at λ_{max} = 832 nm..**30**
Figure S30. Electrochromic switching responses of the cast film of PAI *p*-**8d** on the ITO-glass slide (coated area ~ 0.8 × 2.2 cm^2) in 0.1 M TBAP/MeCN by applying a square-wave potential step between (a) 0.00 V and 0.73 V, monitored at λ_{max} = 430 nm and (b) 0.00 V and 1.10 V monitored at λ_{max} = 828 nm..**31**
Figure S31. Electrochromic switching responses of the cast film of PAI *p*-**8e** on the ITO-glass slide (coated area ~ 0.8 × 2.2 cm^2) in 0.1 M TBAP/MeCN by applying a square-wave potential step between (a) 0.00 V and 0.69 V, monitored at λ_{max} = 429 nm and (b) 0.00 V and 1.10 V monitored at λ_{max} = 831 nm..**31**

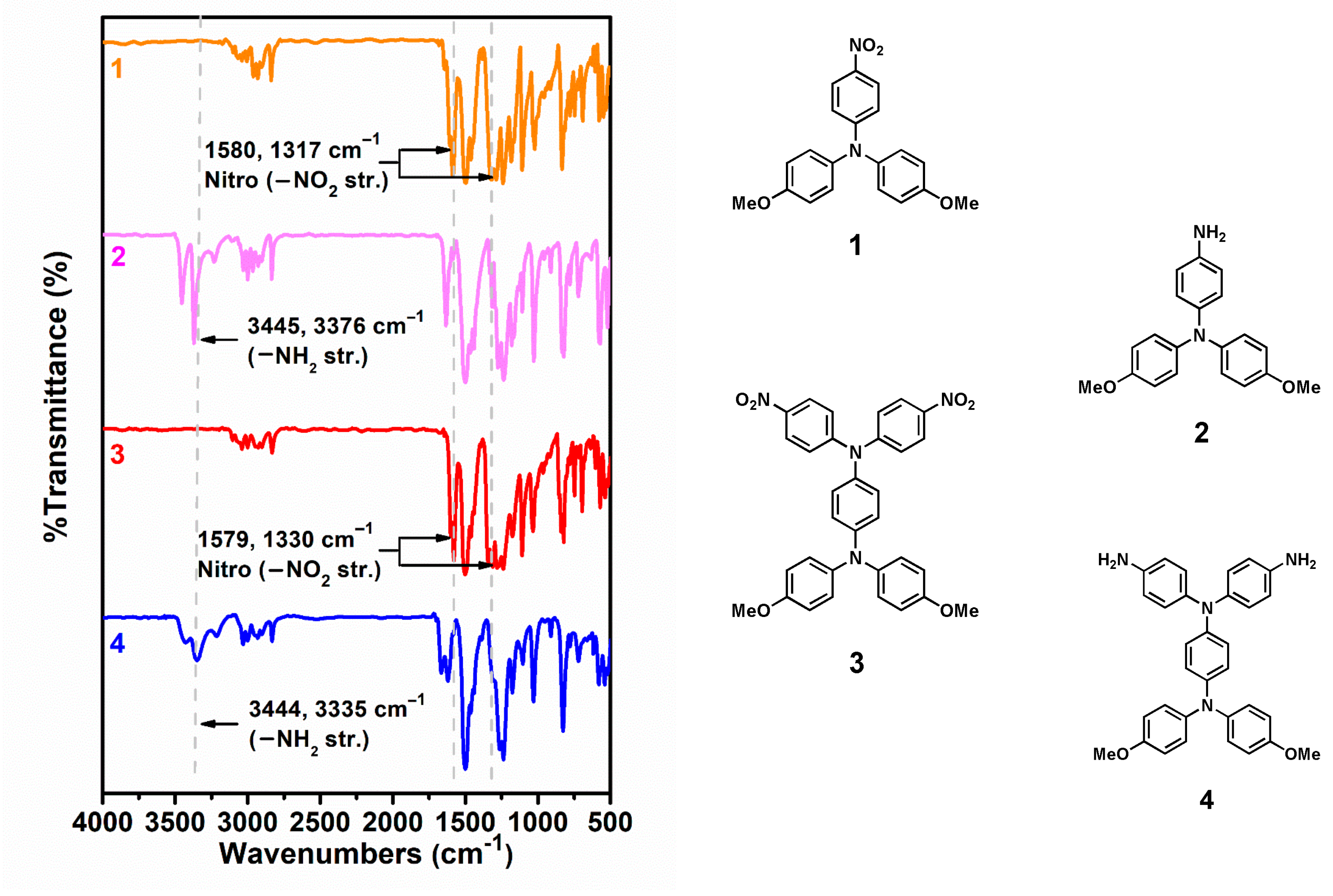

Figure S1. IR spectra of diamine monomer **4** and its precursor compounds.

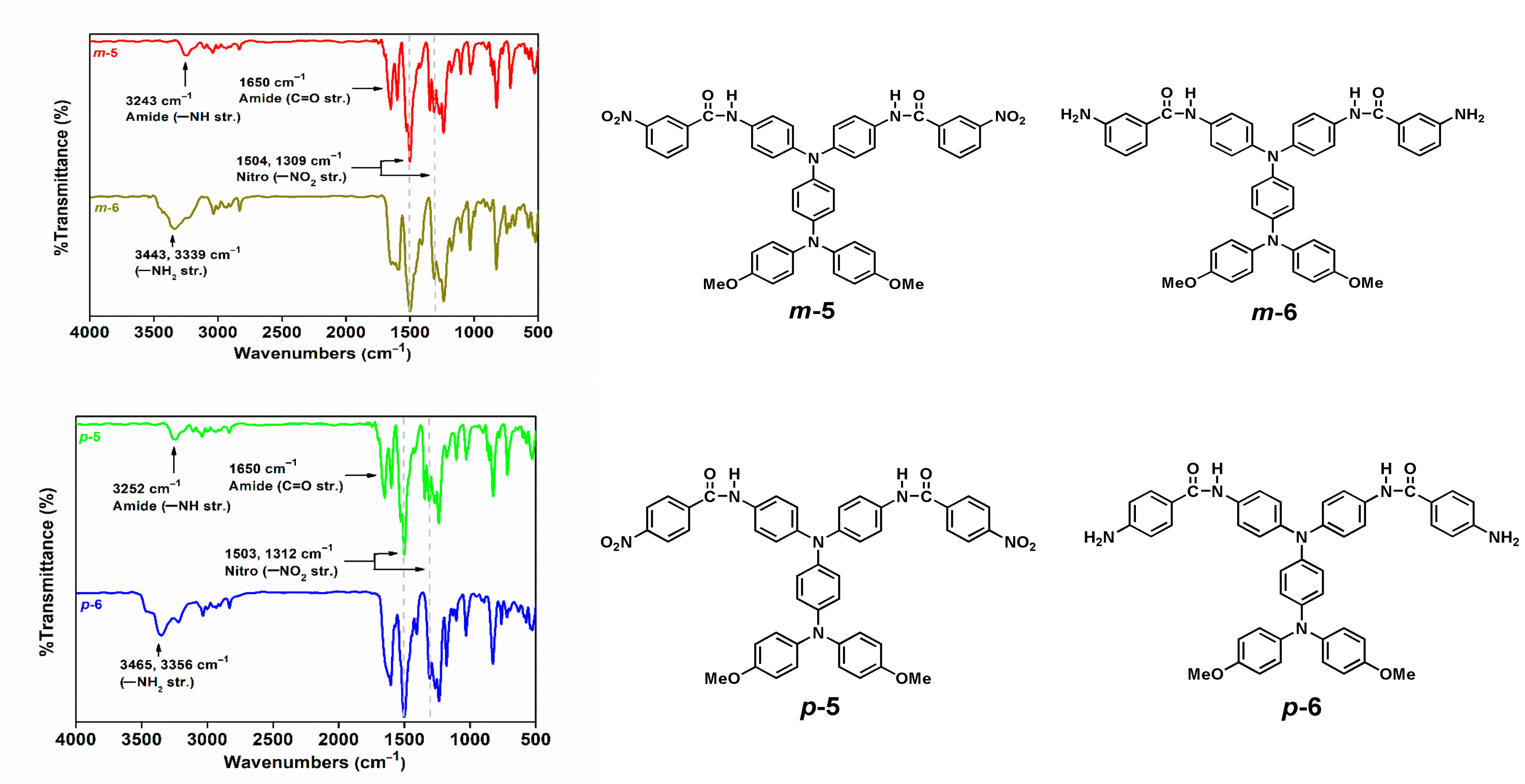

Figure S2. IR spectra of diamide-dinitro compounds ***m*-5** and ***p*-5** and diamide-diamine monomers ***m*-6** and ***p*-6**.

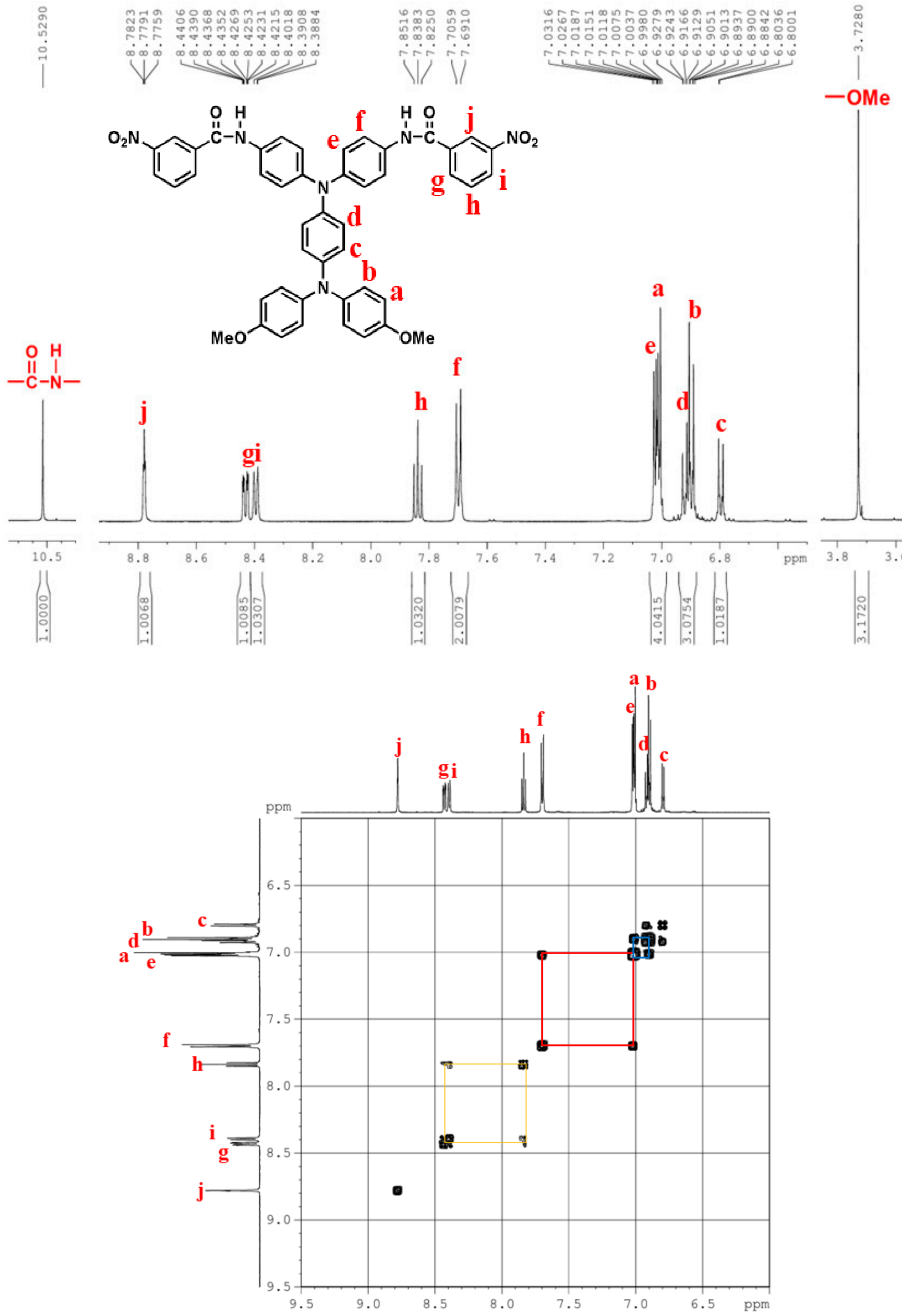

Figure S3. ^{1}H NMR and H-H COSY NMR spectra of diamide-dinitro compound ***m*-5** in DMSO-d_6.

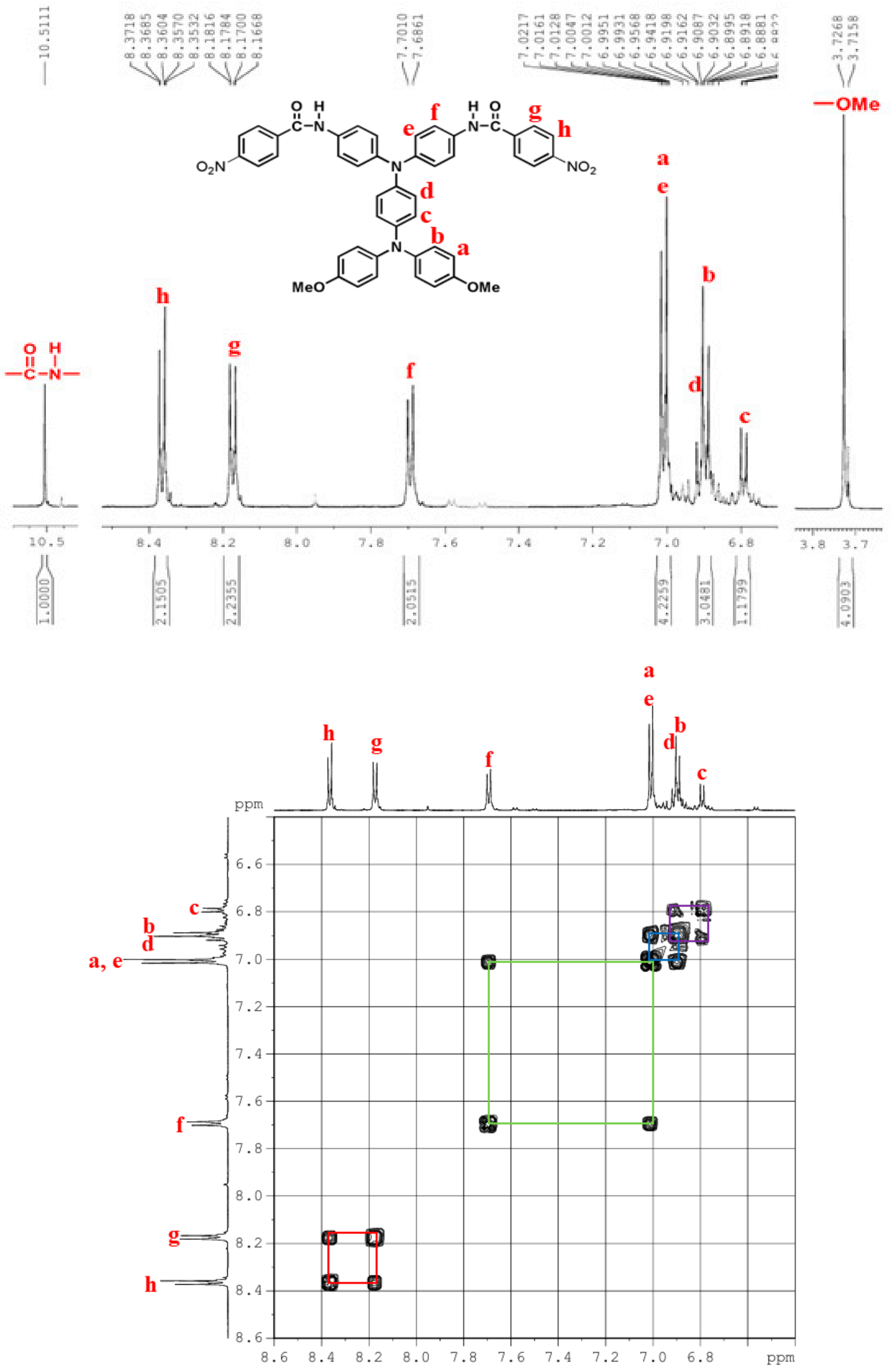

Figure S4. ^{1}H NMR and H-H COSY NMR spectra of diamide-dinitro compound ***p*-5** in DMSO-d_6.

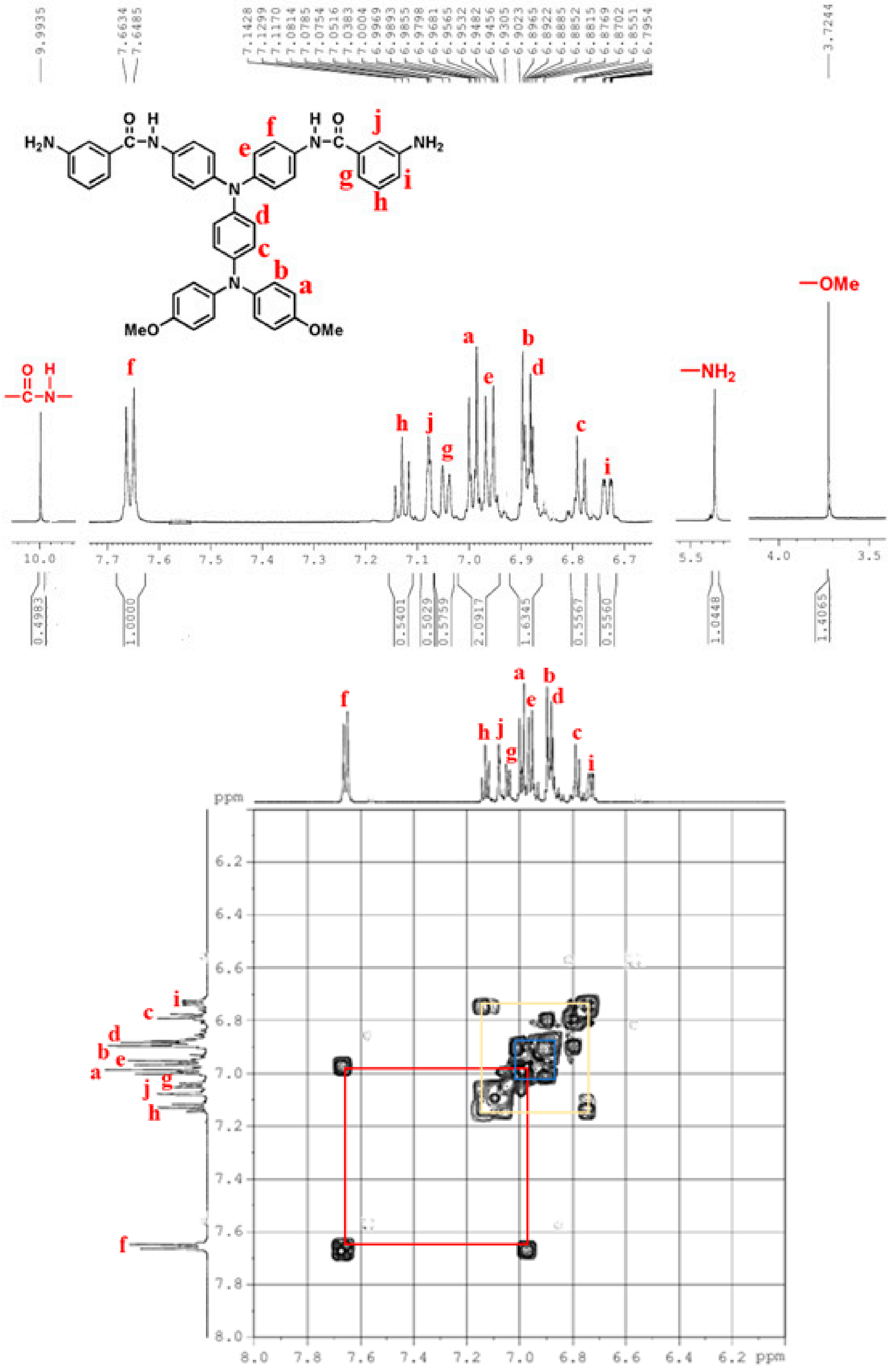

Figure S5. ^{1}H NMR and H-H COSY NMR spectra of diamide-diamine monomer ***m*-6** in DMSO-d_6.

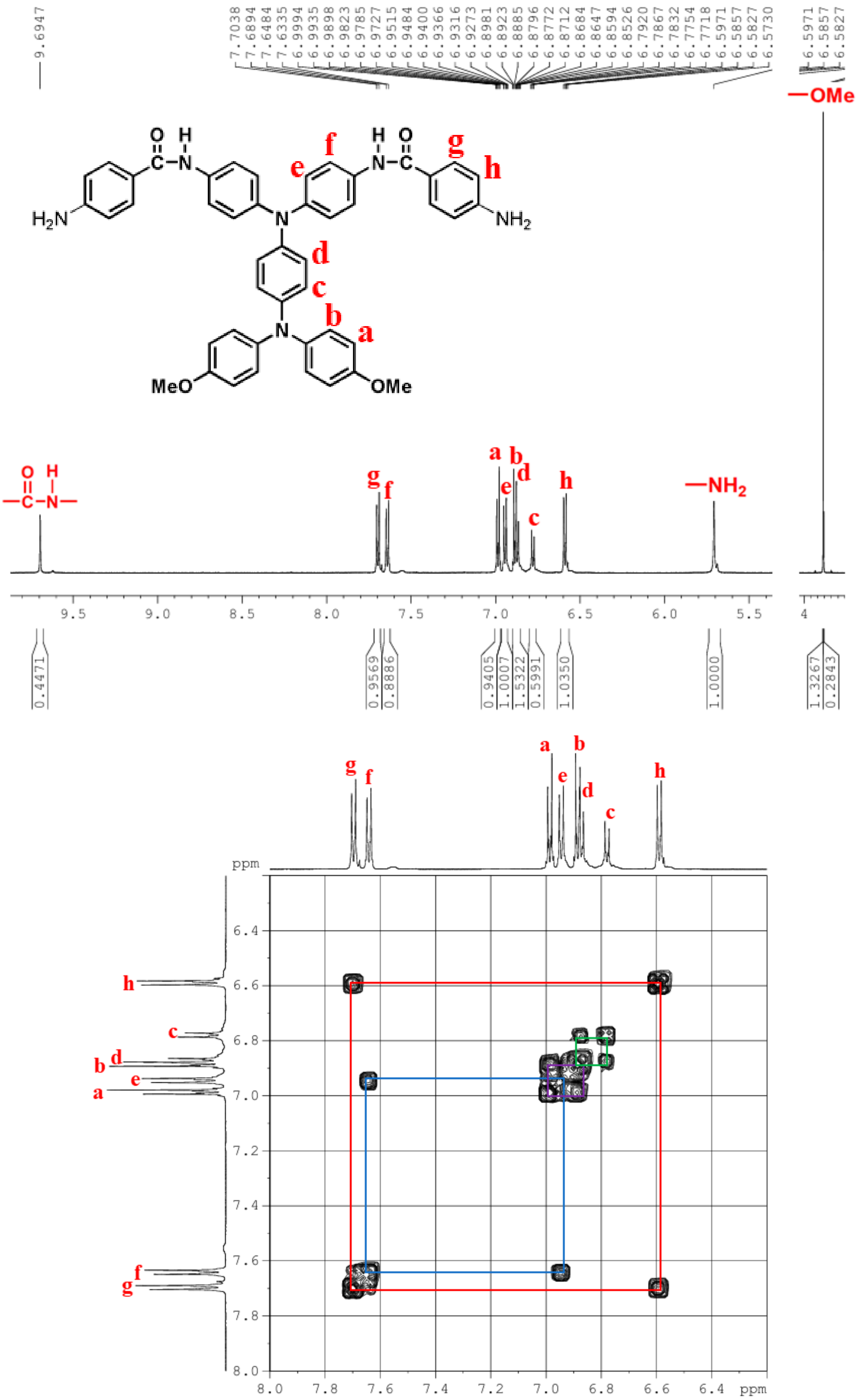

Figure S6.^{1}H NMR and H-H COSY NMR spectra of diamide-diamine monomer ***p*-6** in DMSO-d_6.

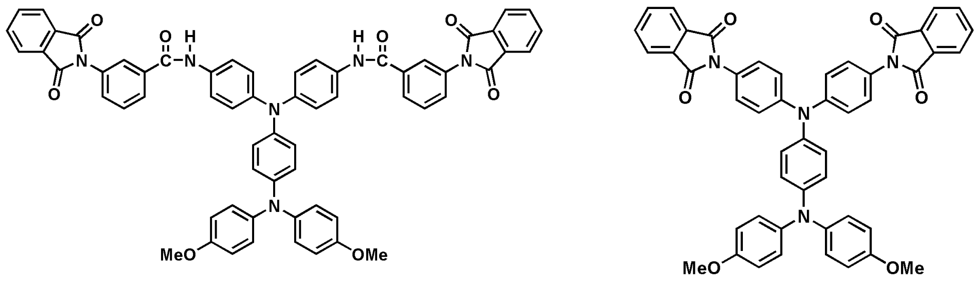

M1 **M2**

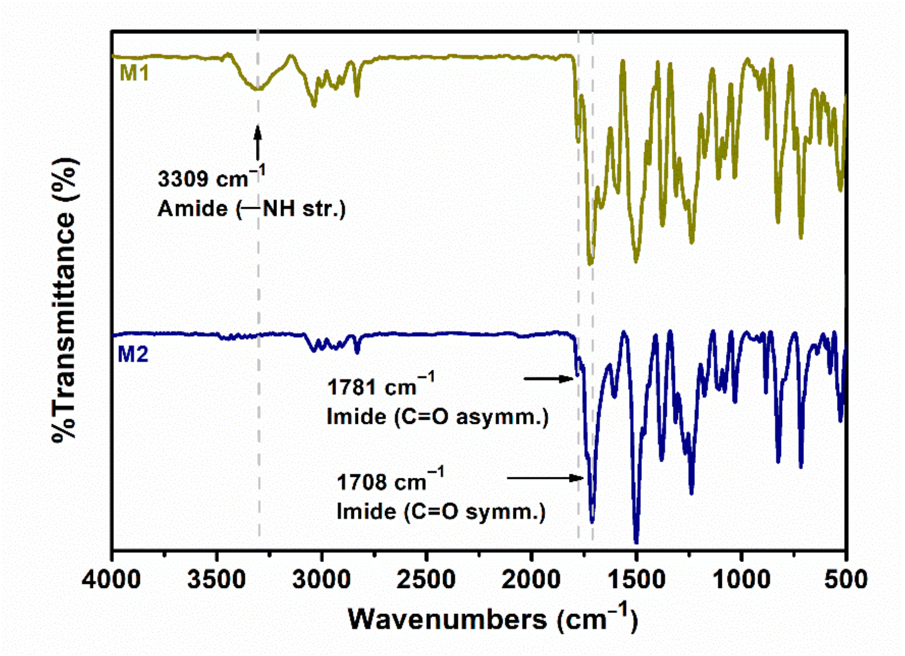

Figure S7. IR spectra of model compounds **M1** and **M2**.

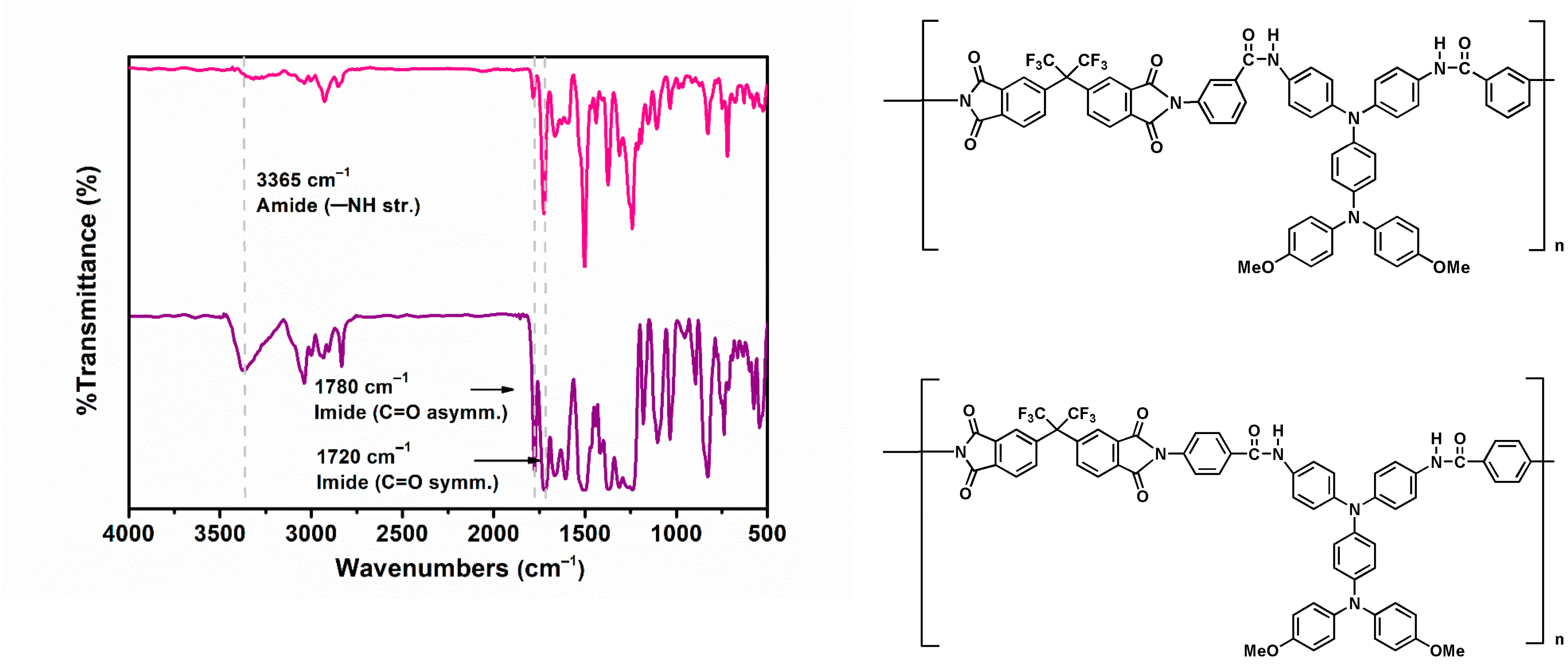

Figure S8. IR spectra of poly(amide-imide)s ***m*-8d** and ***p*-8d**.

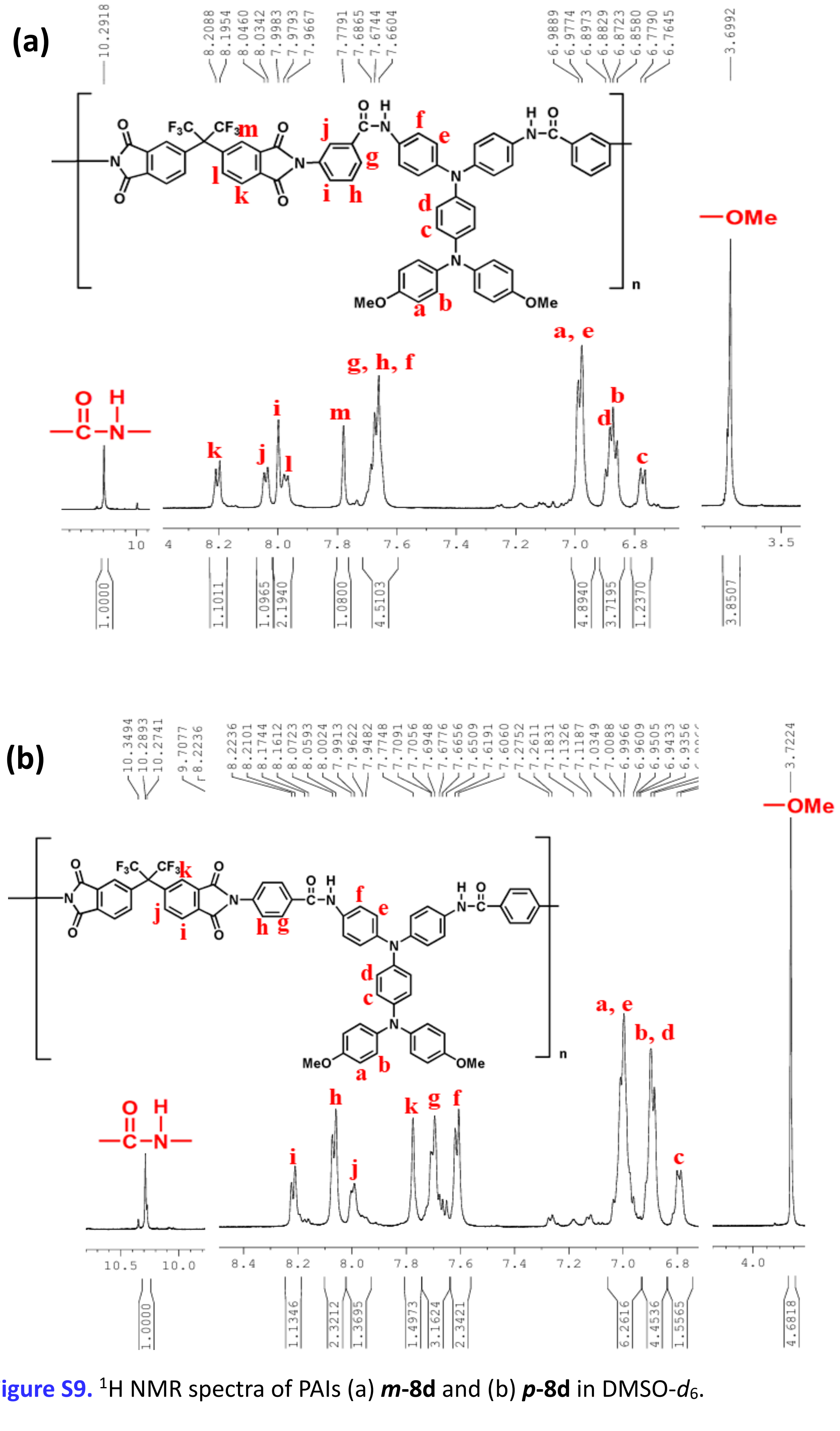

Figure S9. ^{1}H NMR spectra of PAIs (a) ***m*-8d** and (b) ***p*-8d** in DMSO-d_6.

Table S1. Inherent viscosity and GPC data of PAIs

a

Polymer code	η_{inh} (dL/g)[a]	M_n[b]	M_w[b]	PDI[c]
***m*-8a**	0.35	23600	45500	1.93
***m*-8b**	0.44	25200	48000	1.90
***m*-8c**	0.31	23500	45000	1.91
***m*-8d**	0.36	23800	46000	1.93
***m*-8e**	0.85	31700	61000	1.92
***p*-8b**	0.46	24300	47500	1.95
***p*-8c**	0.33	23300	45300	1.94
***p*-8d**	0.42	24100	46500	1.93
***p*-8e**	0.71	29100	56500	1.94

Inherent viscosity measured at a concentration of 0.5 dL/g in DMAc at 30 °C.

[b] Calibrated with polystyrene standards using NMP-0.5 wt% LiCl as eluent at a constant flow rate of 0.6 mL/min at 50 °C.

[c] Polydispersity Index (M_w/M_n).

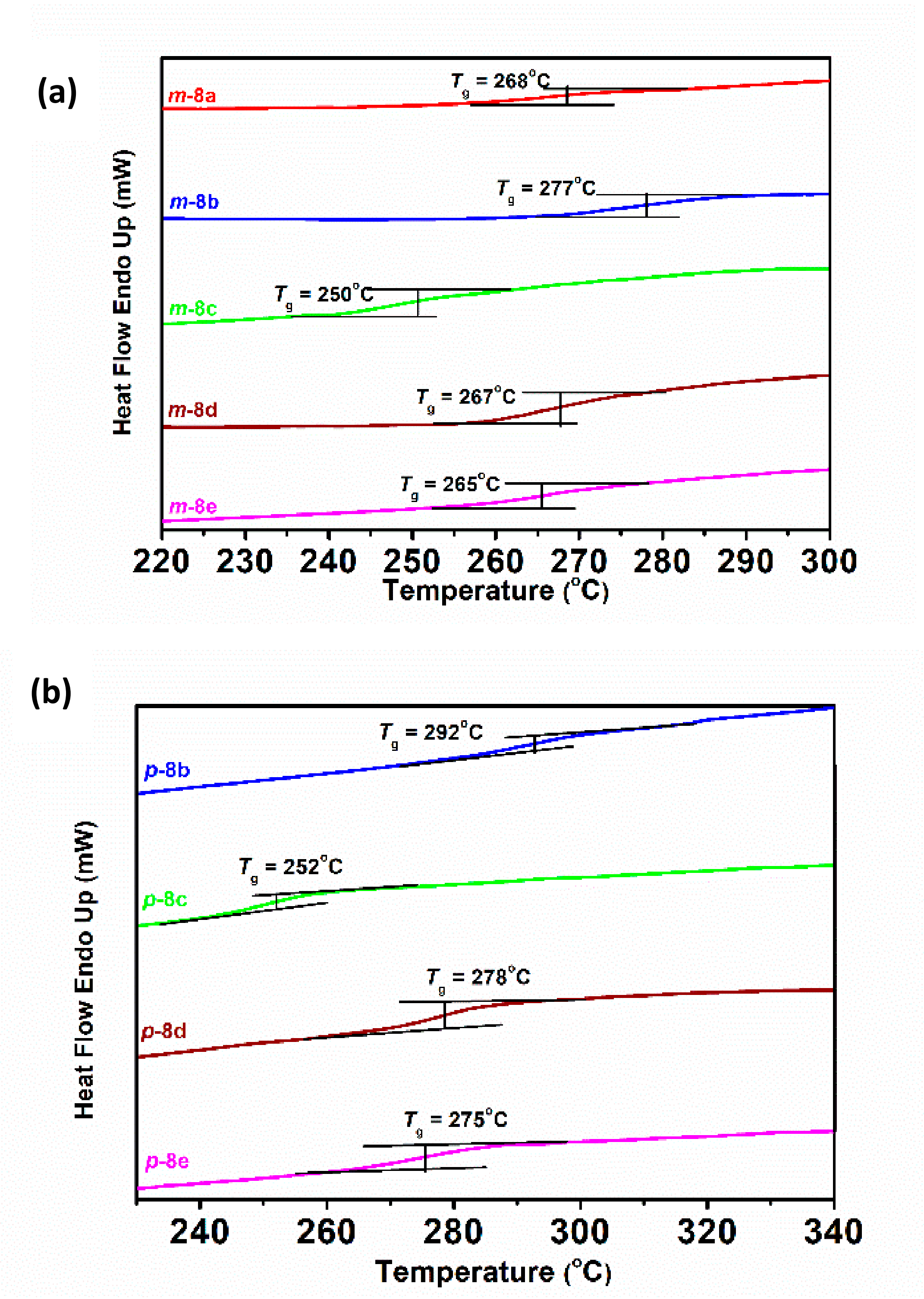

Figure S10. (a) DSC curves of PAIs (a) ***m*-8a** to ***m*-8e** and (b) ***p*-8b** to ***p*-8e** with a heating rate of 20 °C/min in nitrogen.

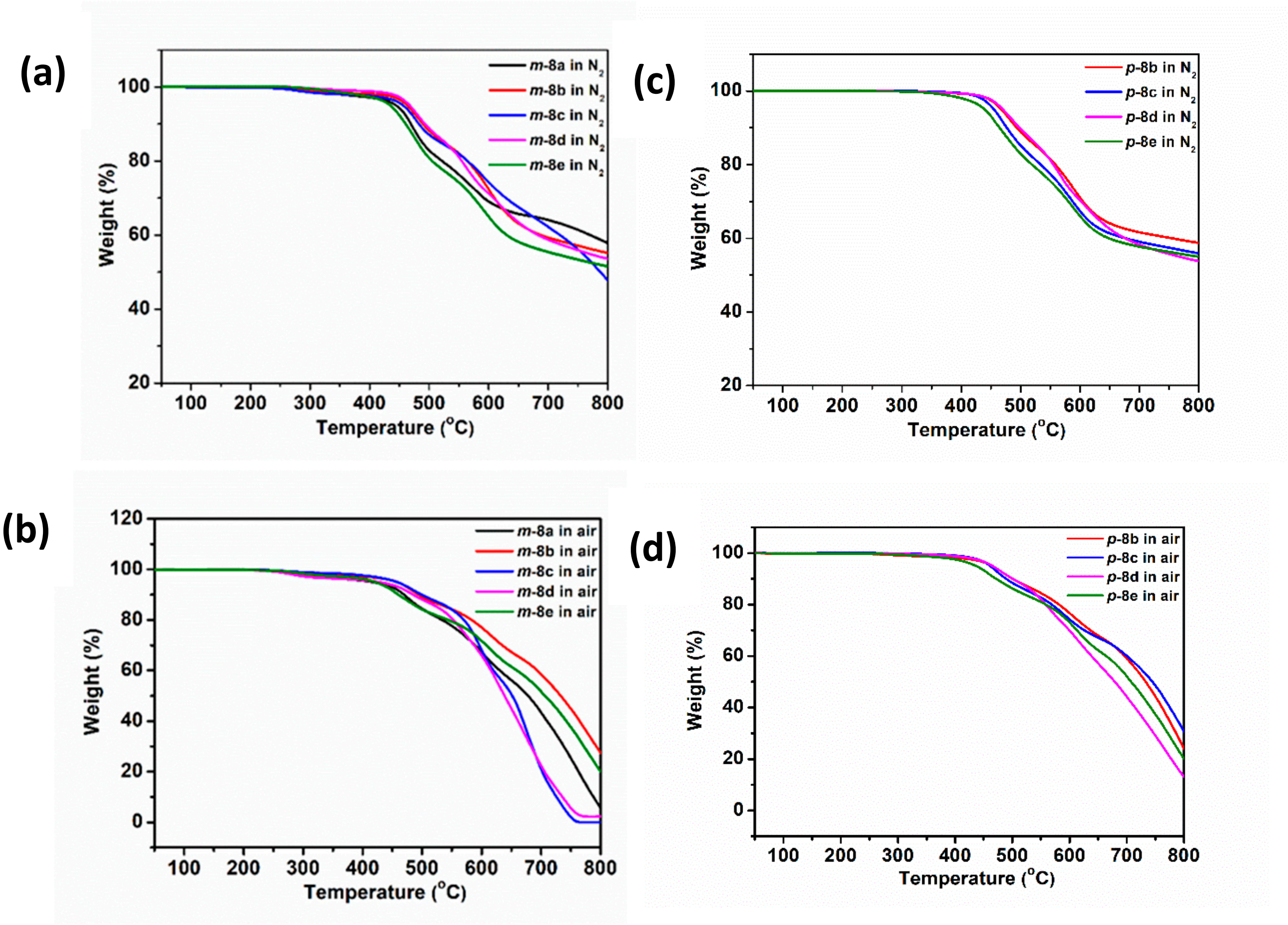

Figure S11. TGA thermograms of PAIs (a) ***m*-8a** to ***m*-8e** in nitrogen, (b) ***m*-8a** to ***m*-8e** in air, (c) ***p*-8b** to ***p*-8e** in nitrogen, and (d) ***p*-8b** to ***p*-8e** in air, with a heating rate of 20 °C/min.

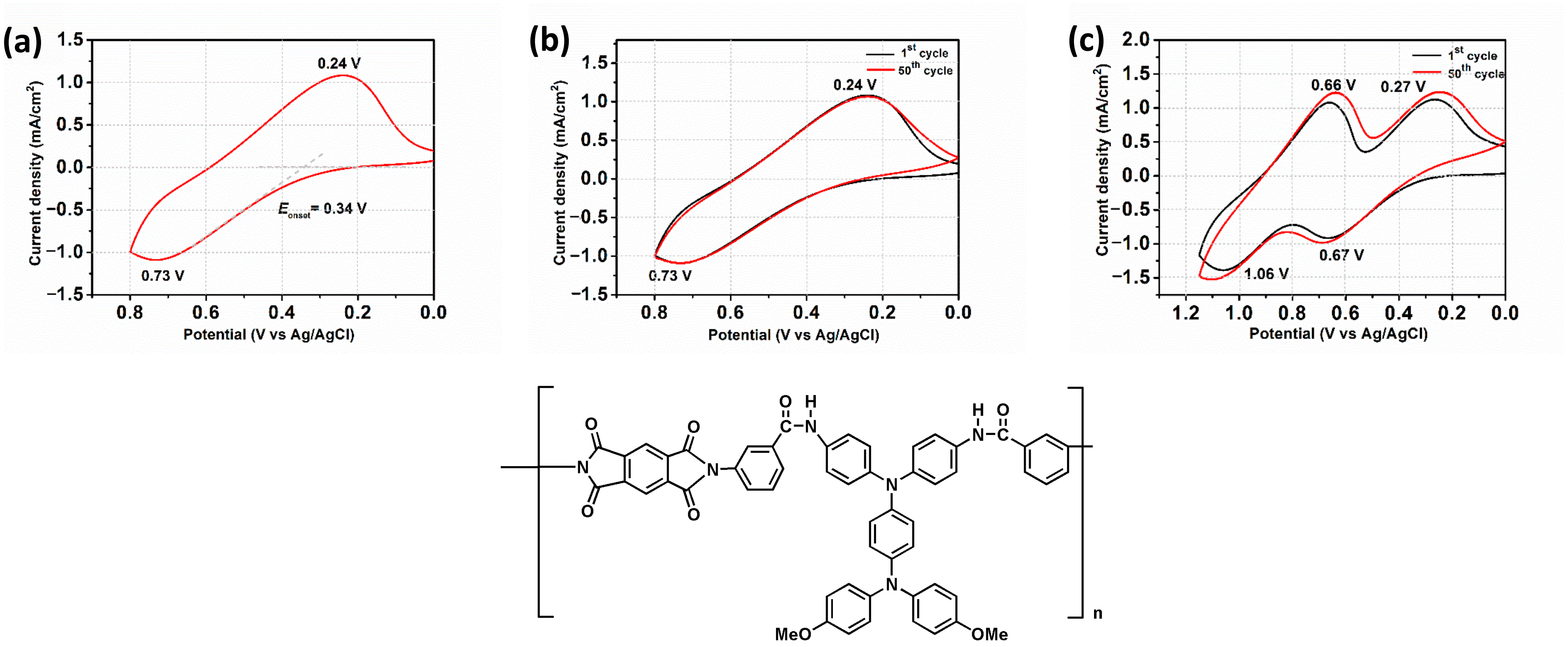

Figure S12. CV diagrams of PAI *m*-**8a** film on ITO-glass slide in 0.1 M Bu_4NClO_4/MeCN at a scan rate of 50 mV/s: (a) first scan in the range of 0–0.8 V, (b) comparison of the first and 50th cycles in the range of 0–0.8 V, and (c) comparison of the first and 50th cycles in the range of 0–1.2 V.

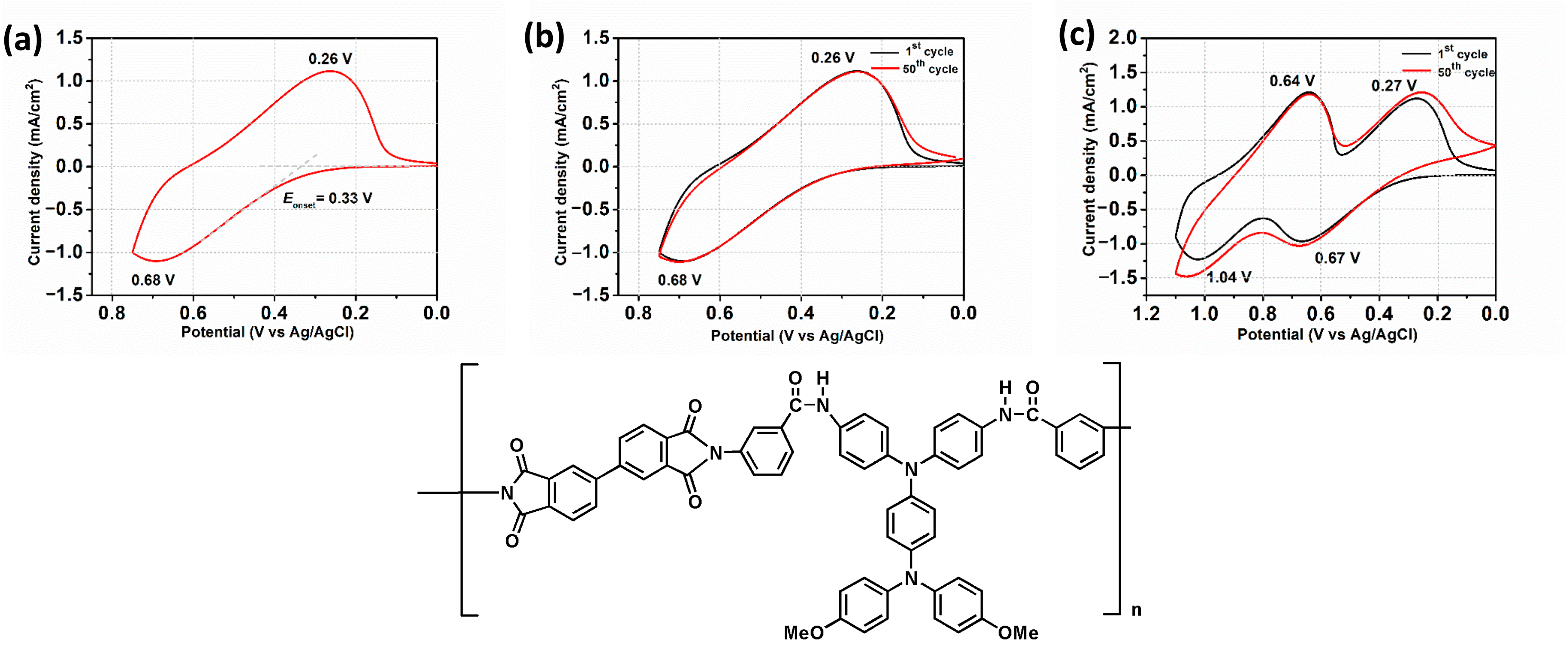

Figure S13. CV diagrams of PAI *m*-**8b** film on ITO-glass slide in 0.1 M Bu_4NClO_4/MeCN at a scan rate of 50 mV/s: (a) first scan in the range of 0–0.8 V, (b) comparison of the first and 50th cycles in the range of 0–0.8 V, and (c) comparison of the first and 50th cycles in the range of 0–1.15 V.

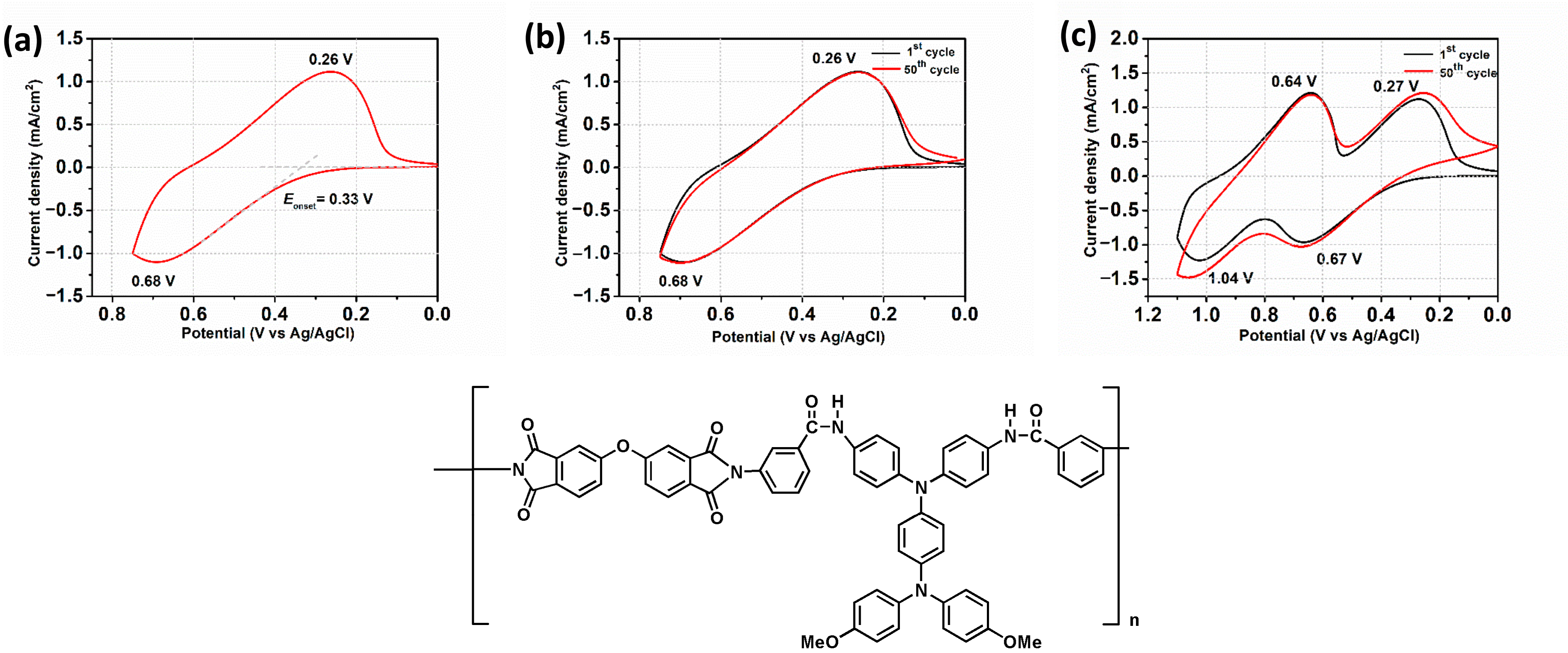

Figure S14. CV diagrams of PAI *m*-**8c** film on ITO-glass slide in 0.1 M Bu_4NClO_4/MeCN at a scan rate of 50 mV/s: (a) first scan in the range of 0–0.75 V, (b) comparison of the first and 50th cycles in the range of 0–0.75 V, and (c) comparison of the first and 50th cycles in the range of 0–1.1 V.

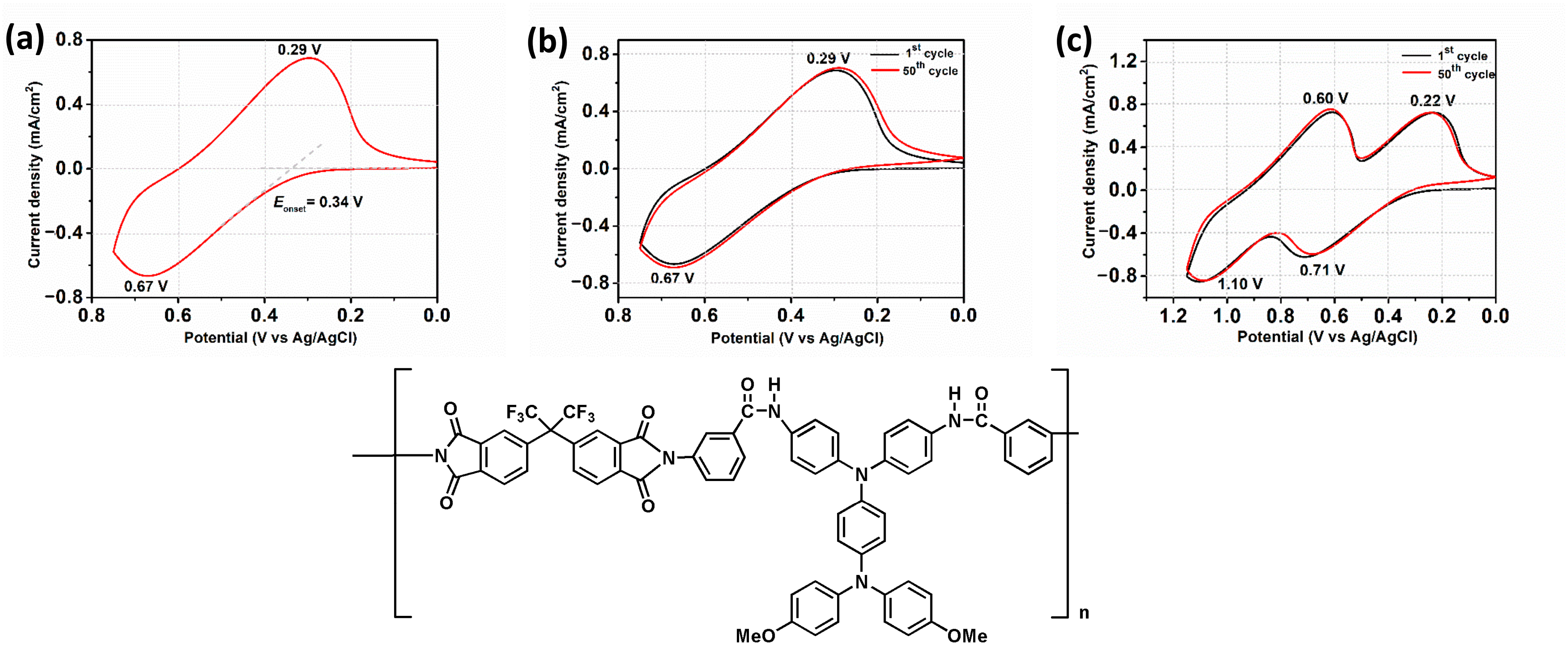

Figure S15. CV diagrams of PAI *m*-**8d** film on ITO-glass slide in 0.1 M Bu_4NClO_4/MeCN at a scan rate of 50 mV/s: (a) first scan in the range of 0–0.8 V, (b) comparison of the first and 50th cycles in the range of 0–0.8 V, and (c) comparison of the first and 50th cycles in the range of 0–1.15 V.

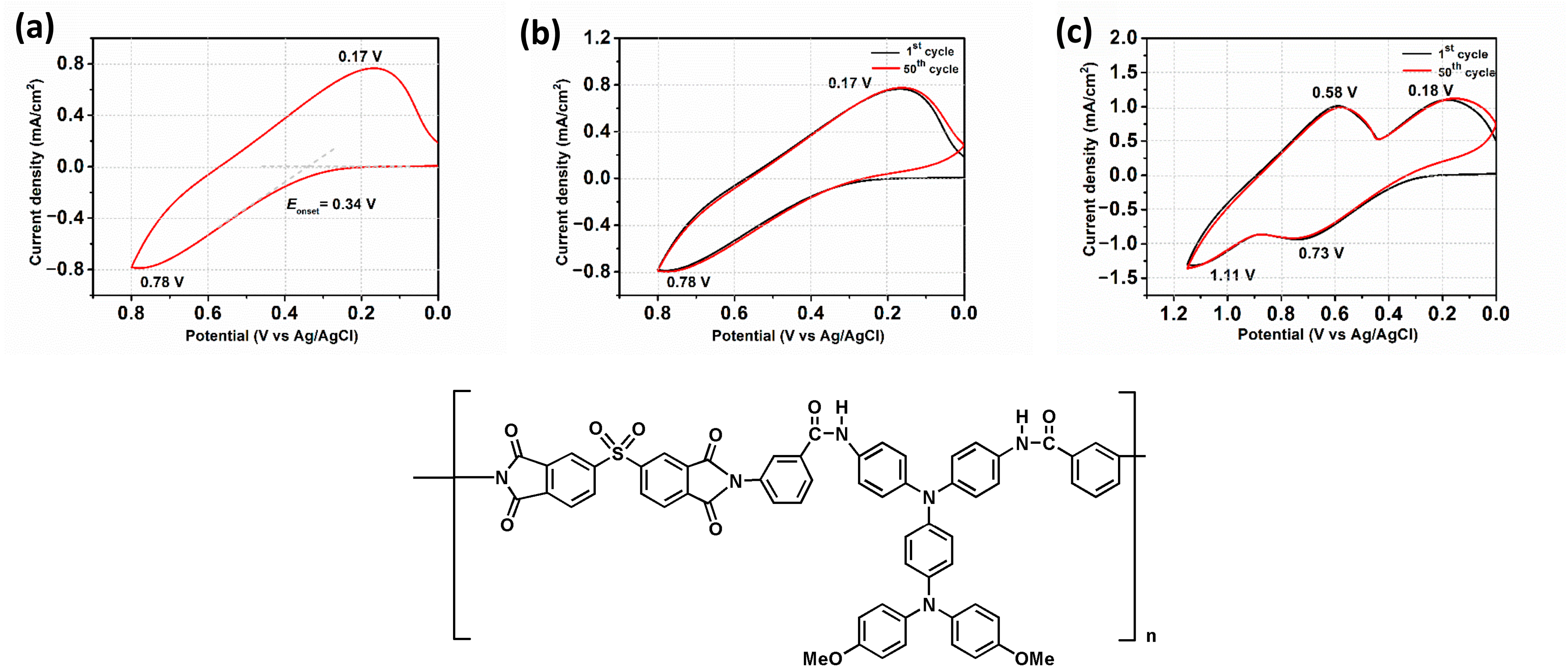

Figure S16. CV diagrams of PAI *m*-**8e** film on ITO-glass slide in 0.1 M Bu_4NClO_4/MeCN at a scan rate of 50 mV/s: (a) first scan in the range of 0–0.8 V, (b) comparison of the first and 50th cycles in the range of 0–0.8 V, and (c) comparison of the first and 50th cycles in the range of 0–1.15 V.

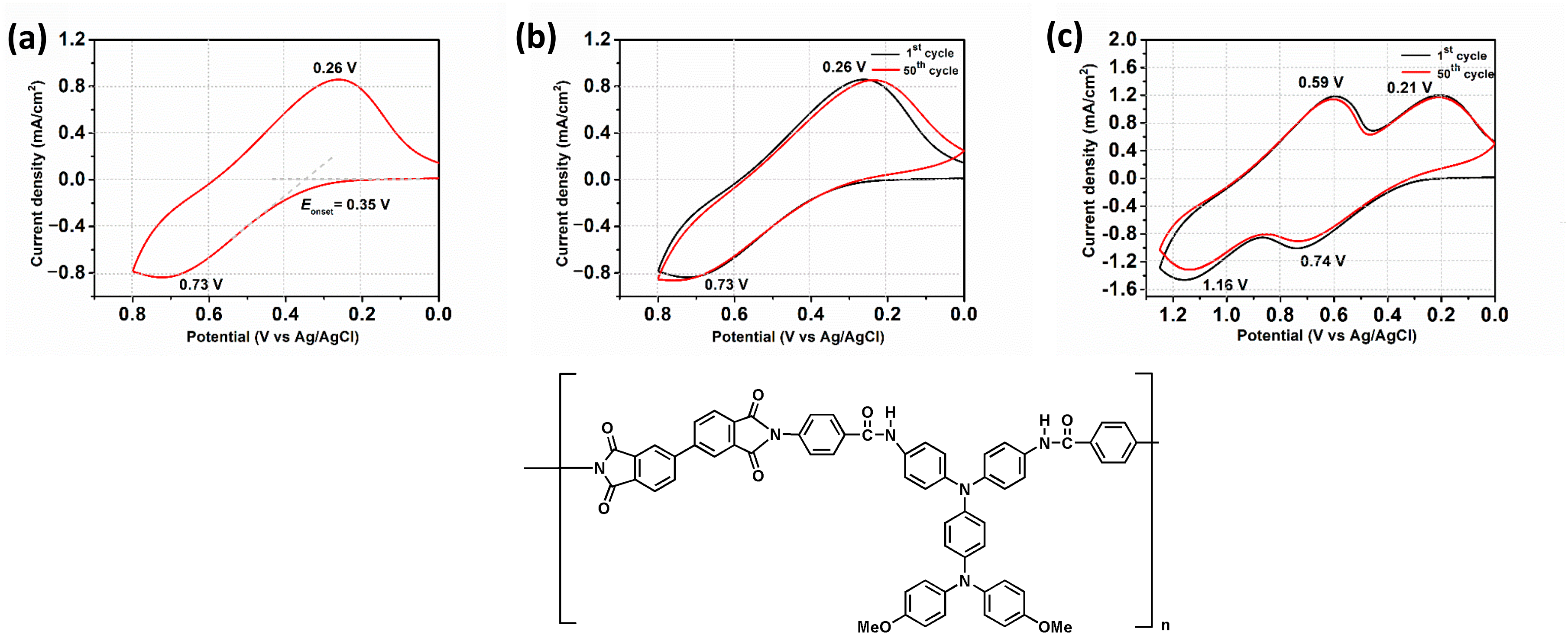

Figure S17. CV diagrams of PAI *p*-**8b** film on ITO-glass slide in 0.1 M Bu_4NClO_4/MeCN at a scan rate of 50 mV/s: (a) first scan in the range of 0–0.8 V, (b) comparison of the first and 50th cycles in the range of 0–0.8 V, and (c) comparison of the first and 50th cycles in the range of 0–1.15 V.

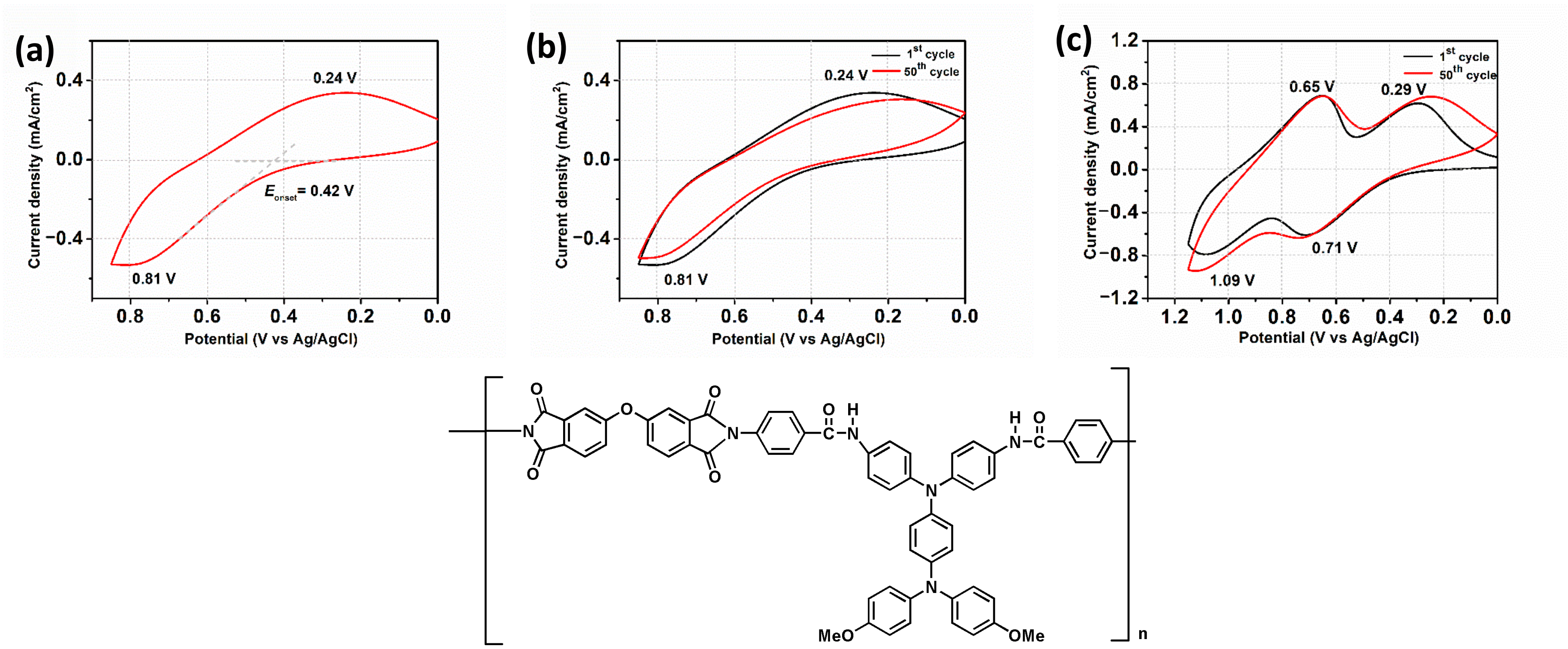

Figure S18. CV diagrams of PAI *p*-**8c** film on ITO-glass slide in 0.1 M Bu_4NClO_4/MeCN at a scan rate of 50 mV/s: (a) first scan in the range of 0–0.85 V, (b) comparison of the first and 50th cycles in the range of 0–0.85 V, and (c) comparison of the first and 50th cycles in the range of 0–1.15 V.

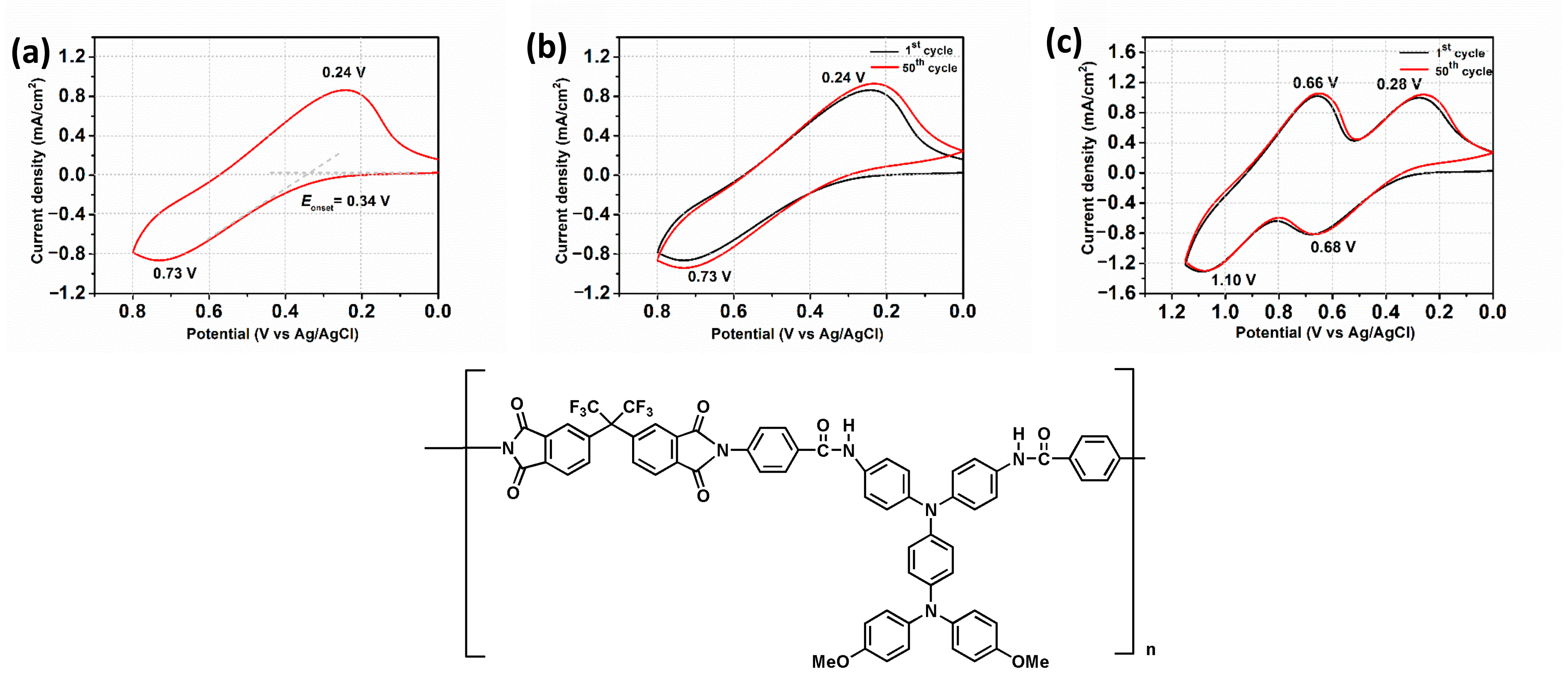

Figure S19. CV diagrams of PAI *p*-**8d** film on ITO-glass slide in 0.1 M Bu_4NClO_4/MeCN at a scan rate of 50 mV/s: (a) first scan in the range of 0–0.8 V, (b) comparison of the first and 50th cycles in the range of 0–0.8 V, and (c) comparison of the first and 50th cycles in the range of 0–1.15 V.

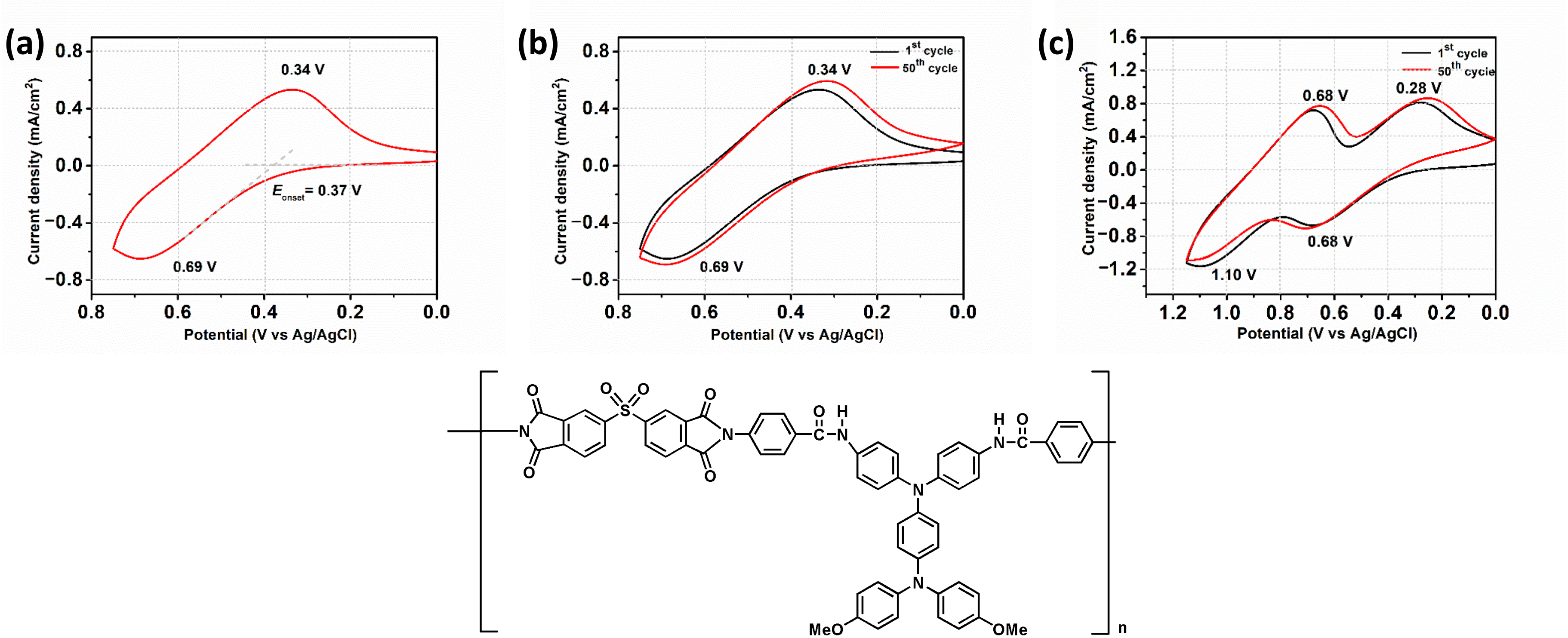

Figure S20. CV diagrams of PAI *p*-**8e** film on ITO-glass slide in 0.1 M Bu_4NClO_4/MeCN at a scan rate of 50 mV/s: (a) first scan in the range of 0–0.75 V, (b) comparison of the first and 50th cycles in the range of 0–0.75 V, and (c) comparison of the first and 50th cycles in the range of 0–1.15 V.

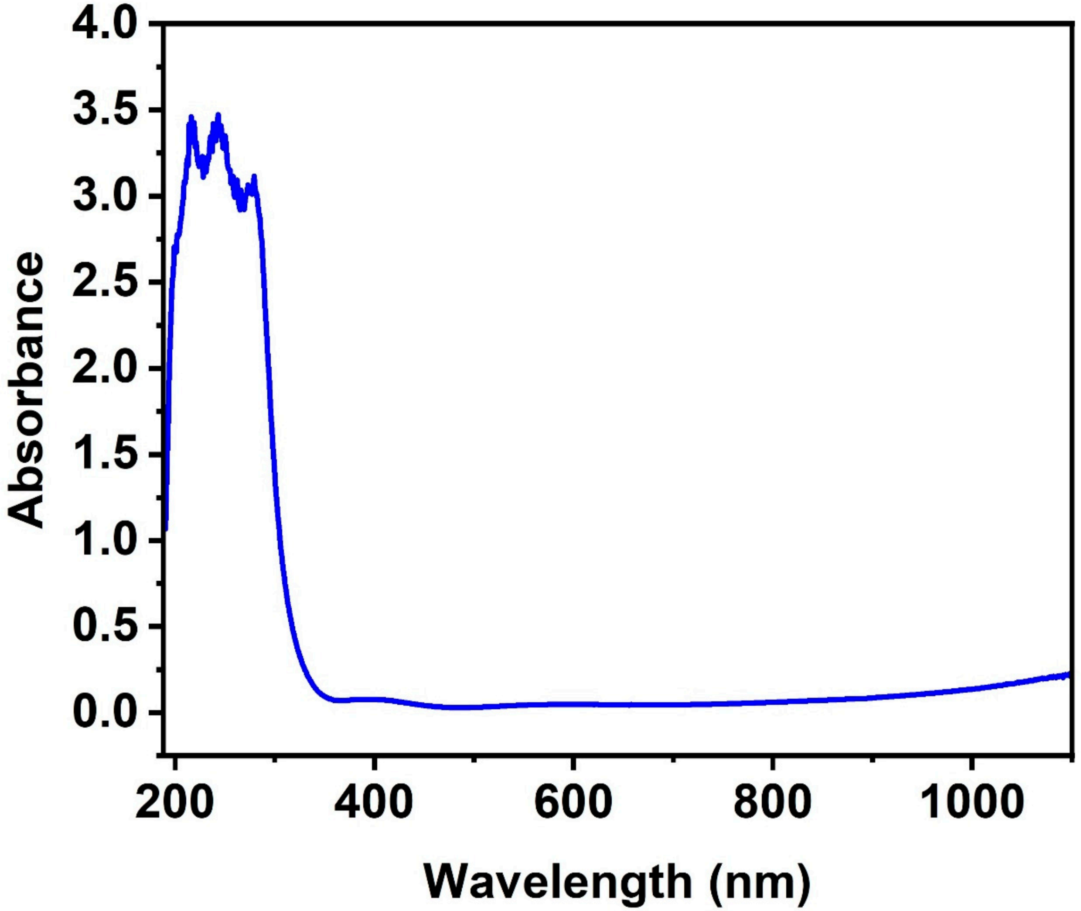

Figure S21. The UV-vis-NIR absorption profile of pure ITO-glass substrate.

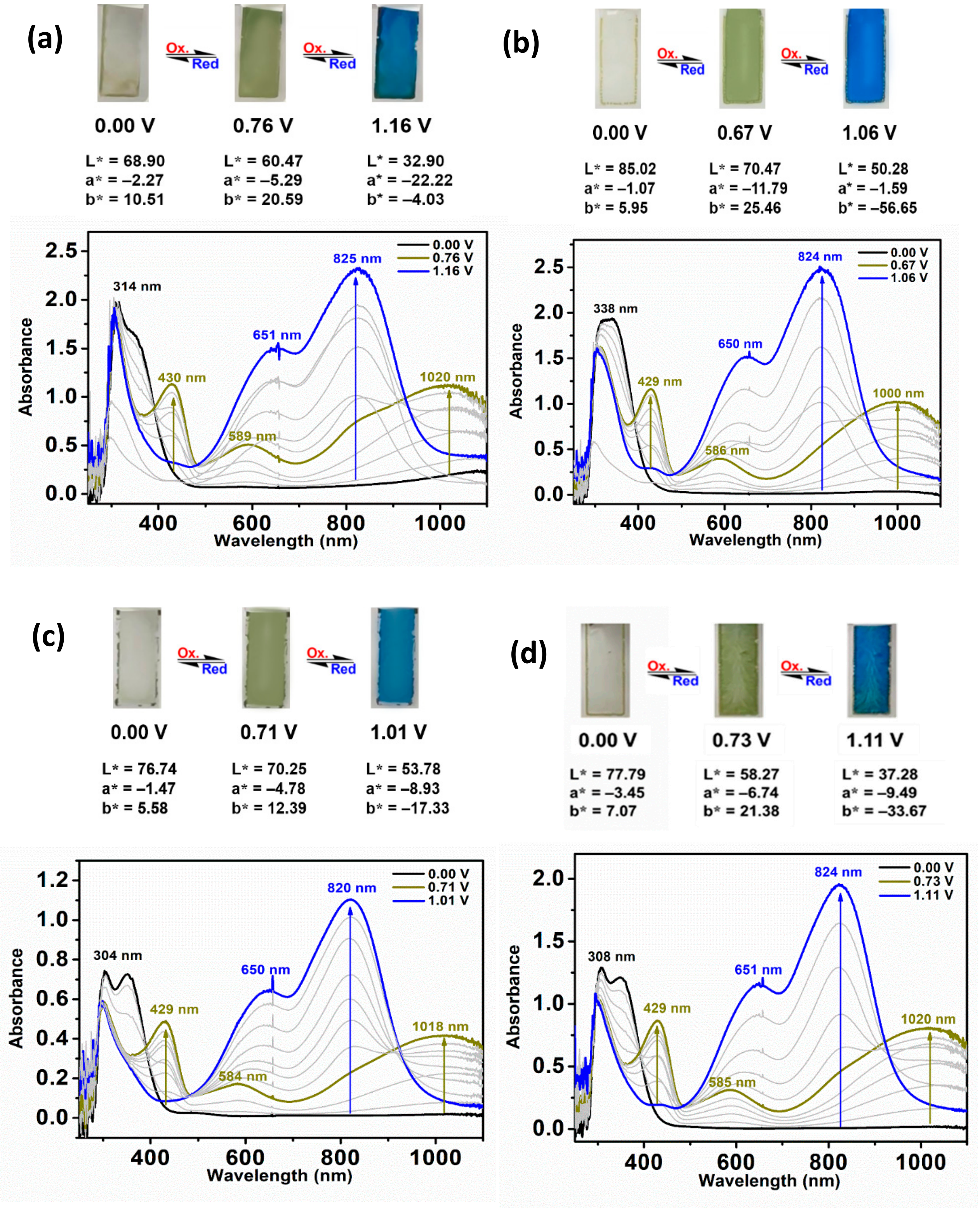

Figure S22. Spectroelectrograms and color changes of the cast films of PAIs (a) ***m*-8a**, (b) ***m*-8b**, (c) ***m*-8d**, and (d) ***m*-8e** on an ITO-glass slide in 0.1 M Bu_4NClO_4/MeCN at various applied voltages.

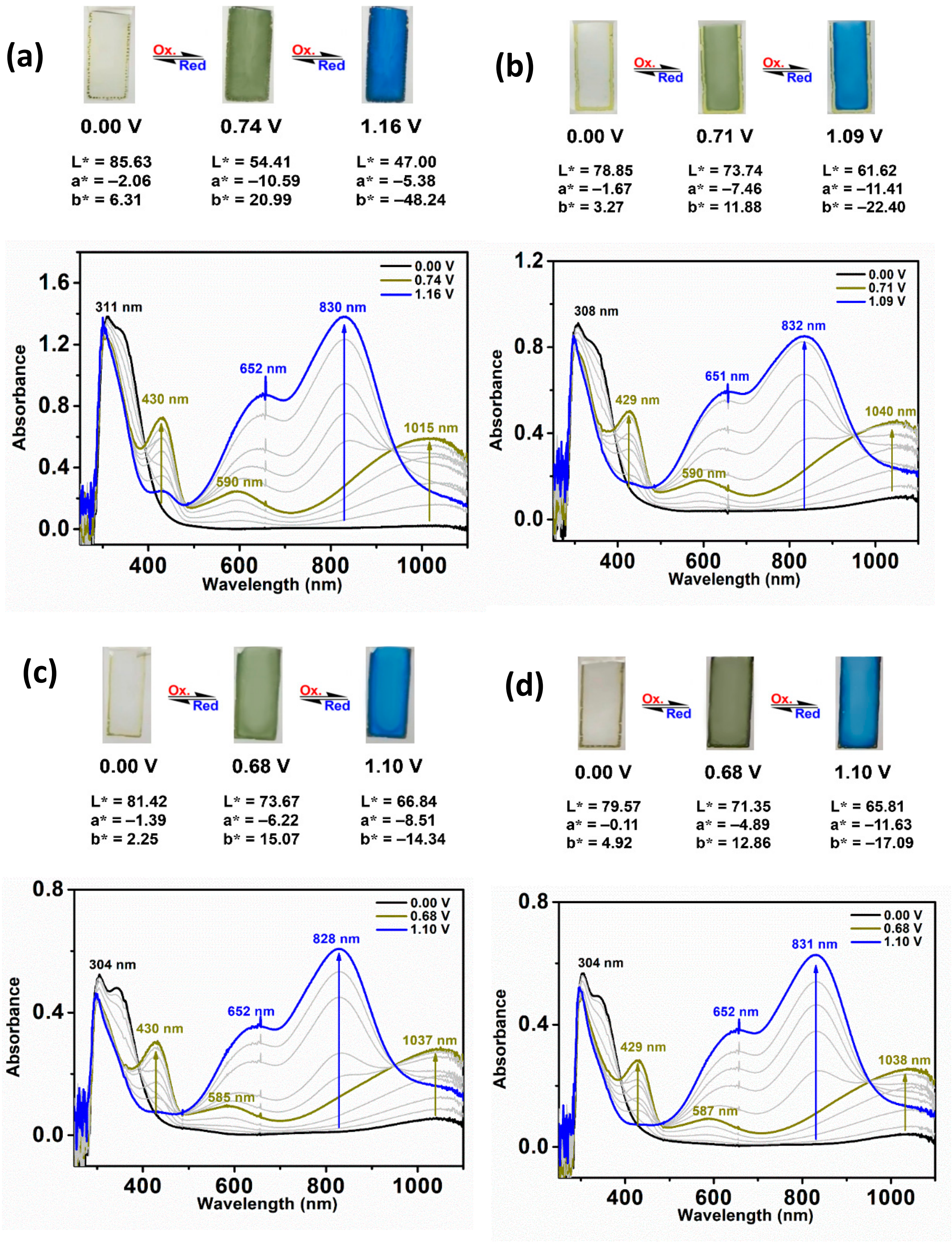

Figure S23. Spectroelectrograms and color changes of the cast films of PAIs (a) ***p*-8b**, (b) ***p*-8c**, (c) ***p*-8d**, and (d) ***p*-8e** on an ITO-glass slide in 0.1 M Bu_4NClO_4/MeCN at various applied voltages.

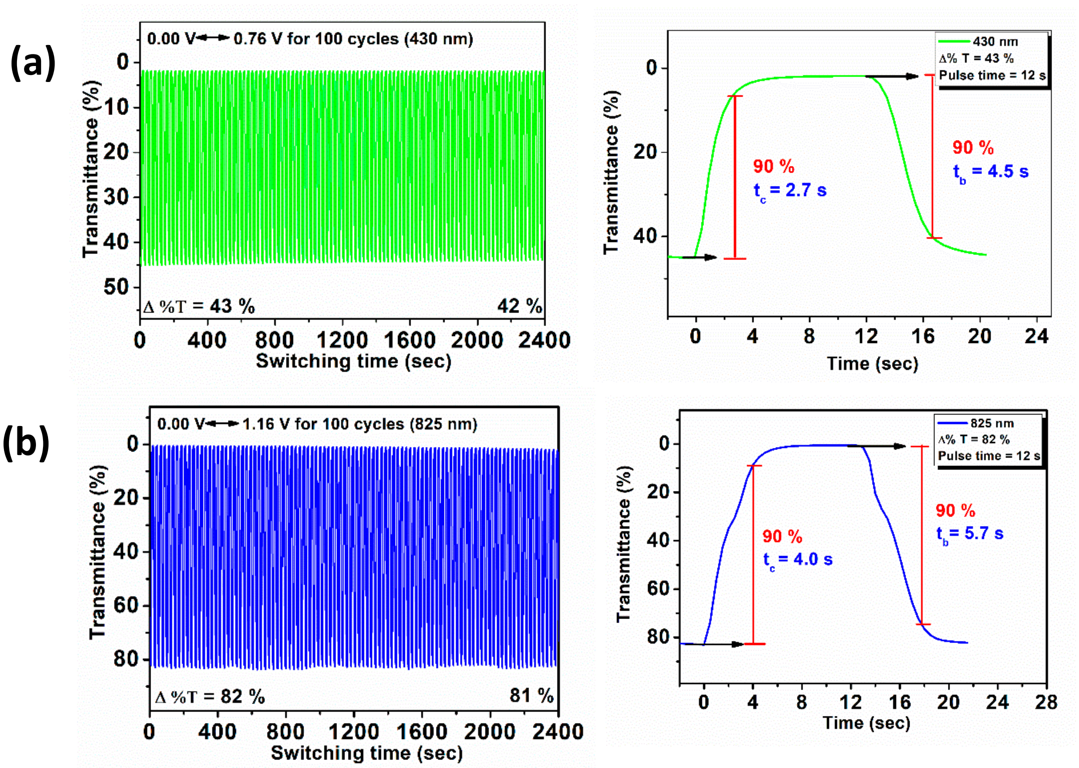

Figure S24. Electrochromic switching responses of the cast film of PAI *m*-**8a** on the ITO-glass slide (coated area ~ 0.8 × 2.2 cm^2) in 0.1 M Bu_4NClO_4 (TBAP)/MeCN by applying a square-wave potential step between (a) 0.00 V and 0.76 V, monitored at λ_{max} = 430 nm and (b) 0.00 V and 1.16 V monitored at λ_{max} = 825 nm.

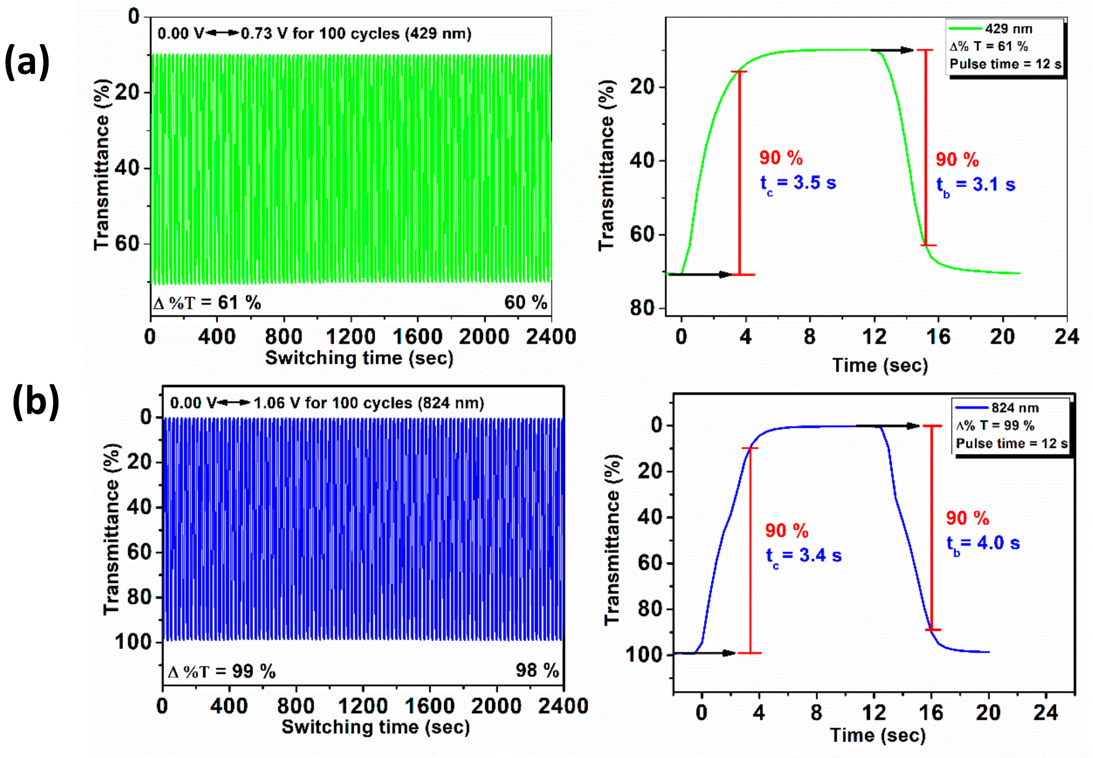

Figure S25. Electrochromic switching responses of the cast film of PAI *m*-**8b** on the ITO-glass slide (coated area ~ 0.8 × 2.2 cm^2) in 0.1 M TBAP/MeCN by applying a square-wave potential step between (a) 0.00 V and 0.73 V, monitored at λ_{max} = 429 nm and (b) 0.00 V and 1.06 V monitored at λ_{max} = 824 nm.

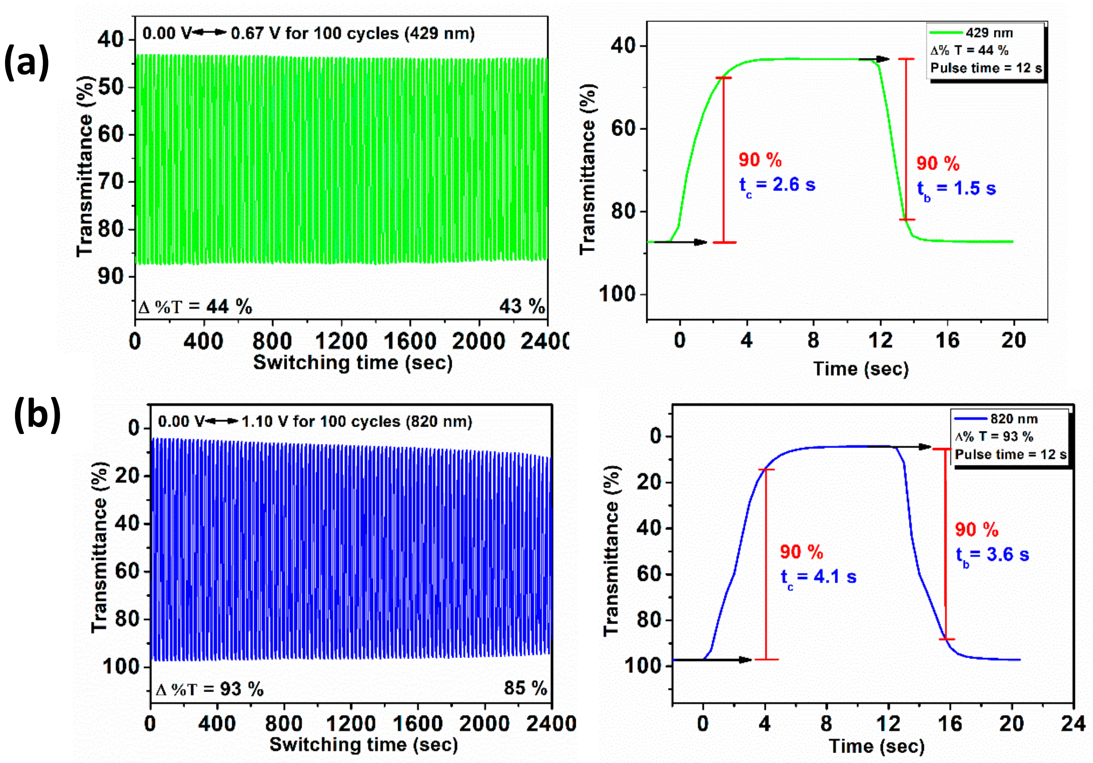

Figure 26. Electrochromic switching responses of the cast film of PAI *m*-**8d** on the ITO-glass slide (coated area ~ 0.8 × 2.2 cm^2) in 0.1 M TBAP/MeCN by applying a square-wave potential step between (a) 0.00 V and 0.67 V, monitored at λ_{max} = 429 nm and (b) 0.00 V and 1.10 V monitored at λ_{max} = 820 nm.

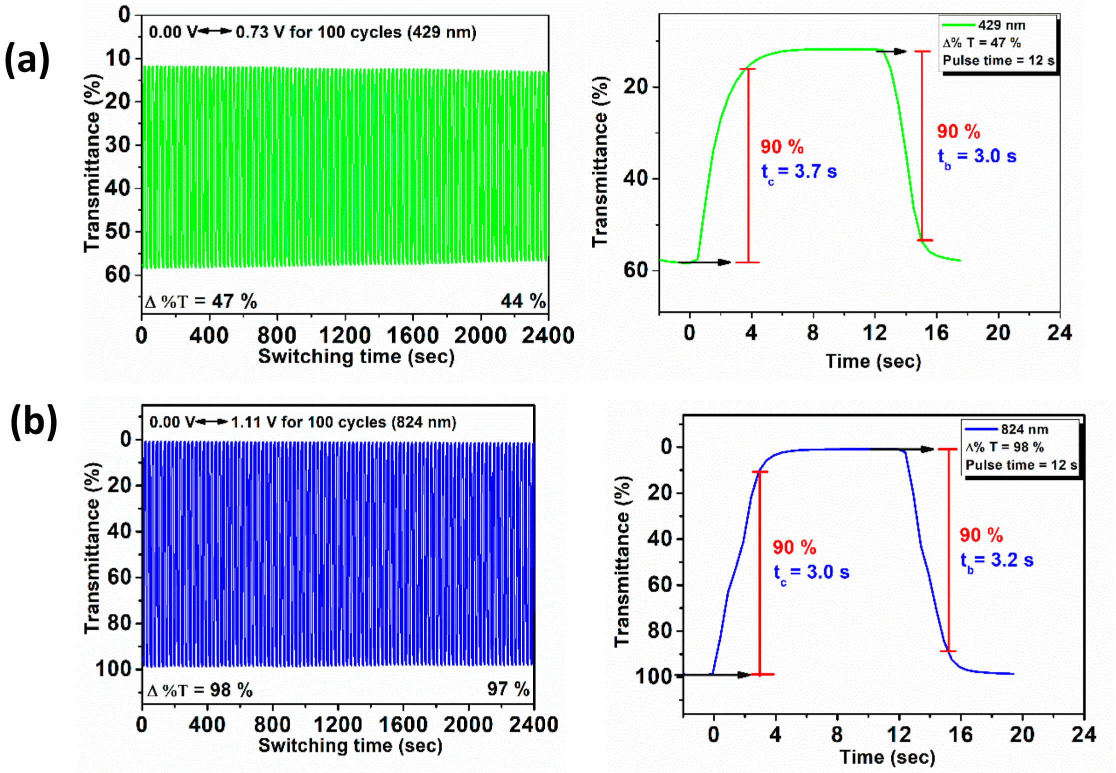

Figure S27. Electrochromic switching responses of the cast film of PAI *m*-**8e** on the ITO-glass slide (coated area ~ 0.8 × 2.2 cm^2) in 0.1 M TBAP/MeCN by applying a square-wave potential step between (a) 0.00 V and 0.73 V, monitored at λ_{max} = 429 nm and (b) 0.00 V and 1.11 V monitored at λ_{max} = 824 nm.

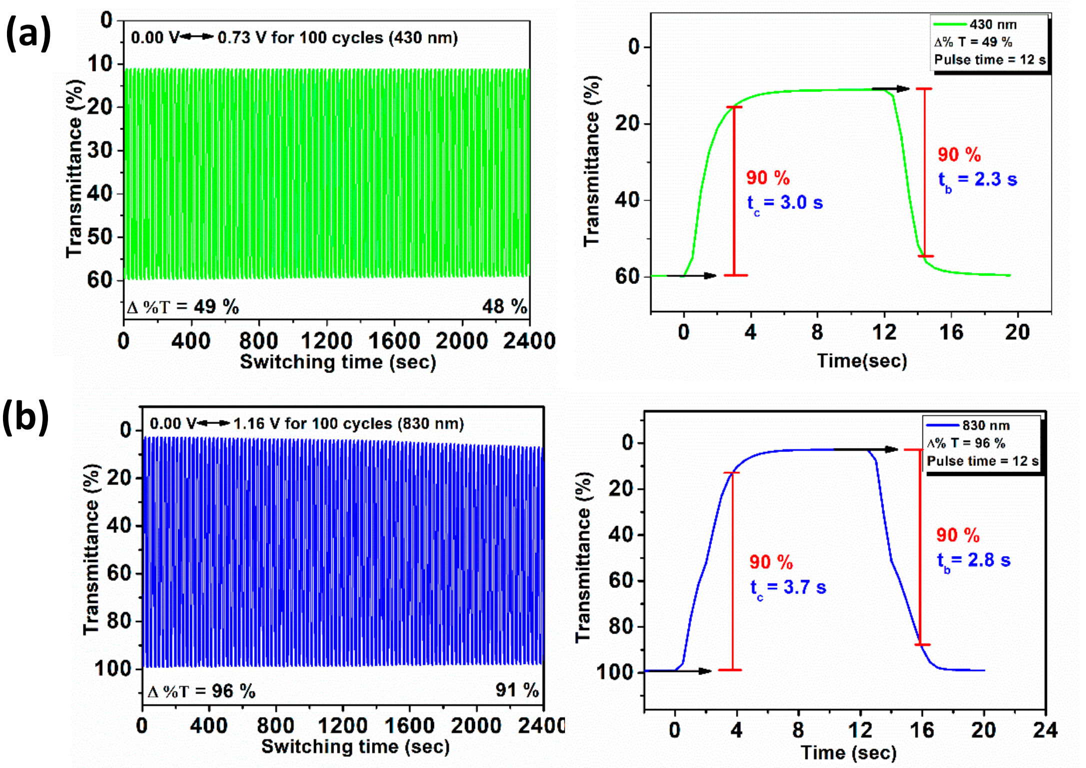

Figure S28. Electrochromic switching responses of the cast film of PAI *p*-**8b** on the ITO-glass slide (coated area ~ 0.8 × 2.2 cm^2) in 0.1 M TBAP/MeCN by applying a square-wave potential step between (a) 0.00 V and 0.73 V, monitored at λ_{max} = 430 nm and (b) 0.00 V and 1.16 V monitored at λ_{max} = 830 nm.

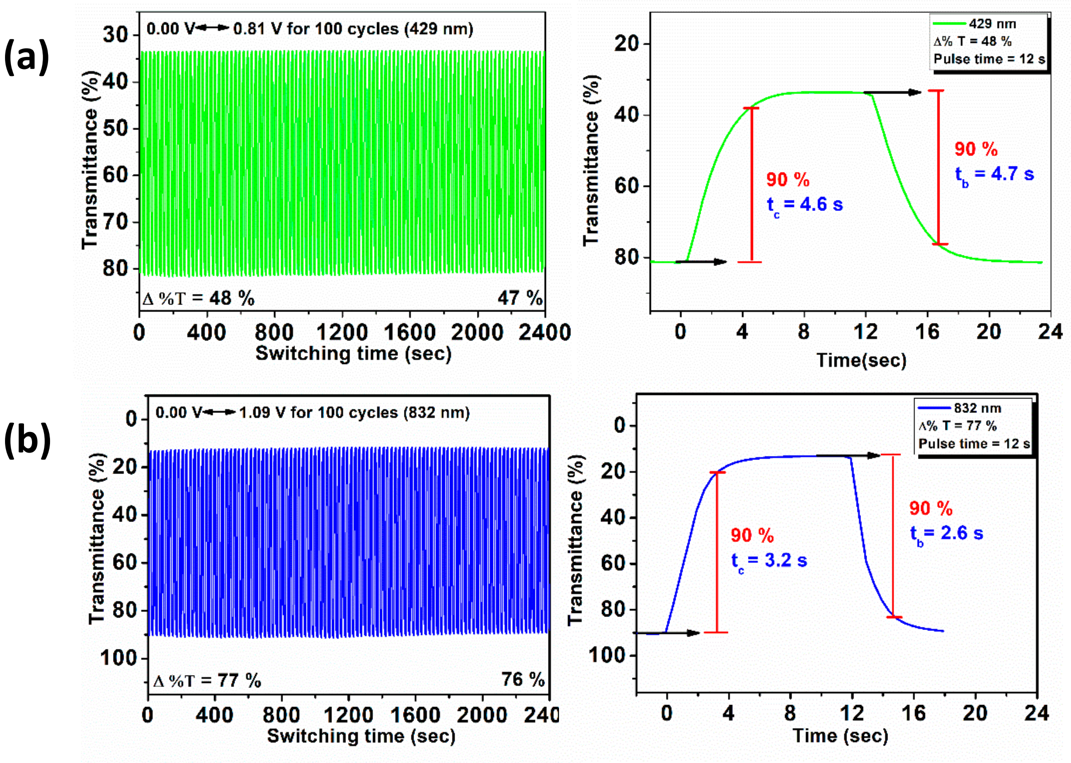

Figure S29. Electrochromic switching responses of the cast film of PAI *p*-**8c** on the ITO-glass slide (coated area ~ 0.8 × 2.2 cm^2) in 0.1 M TBAP/MeCN by applying a square-wave potential step between (a) 0.00 V and 0.81 V, monitored at λ_{max} = 429 nm and (b) 0.00 V and 1.09 V monitored at λ_{max} = 832 nm.

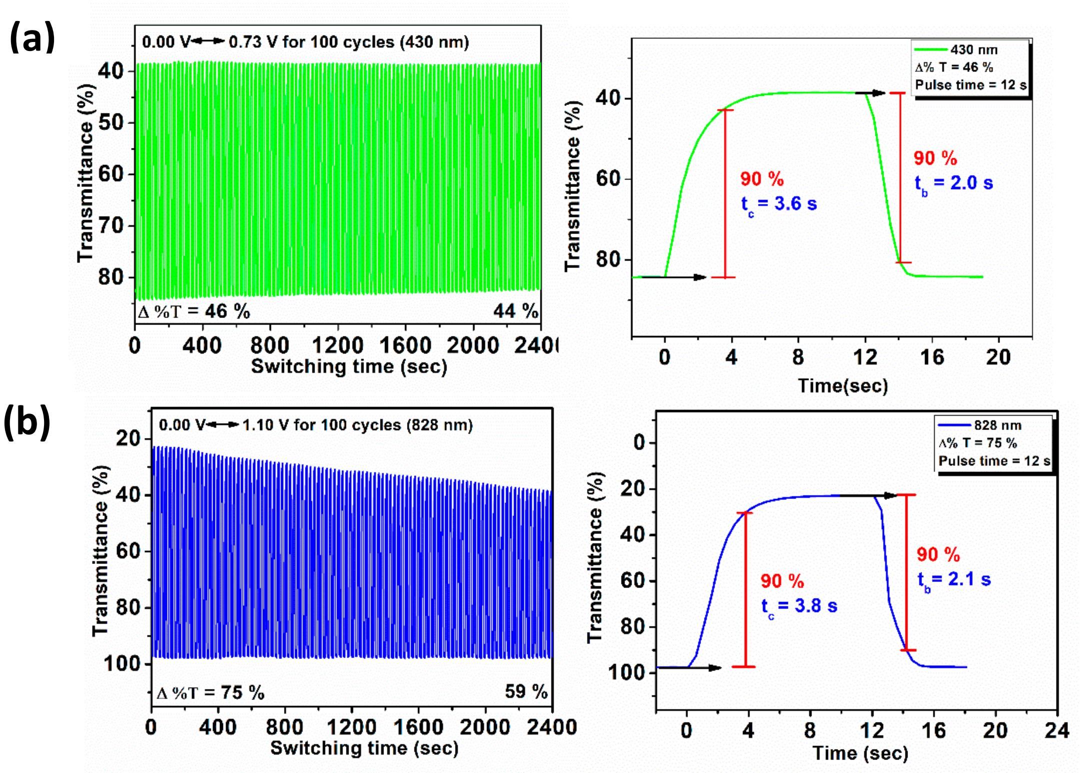

Figure S30. Electrochromic switching responses of the cast film of PAI *p*-**8d** on the ITO-glass slide (coated area ~ 0.8 × 2.2 cm^2) in 0.1 M TBAP/MeCN by applying a square-wave potential step between (a) 0.00 V and 0.73 V, monitored at λ_{max} = 430 nm and (b) 0.00 V and 1.10 V monitored at λ_{max} = 828 nm.

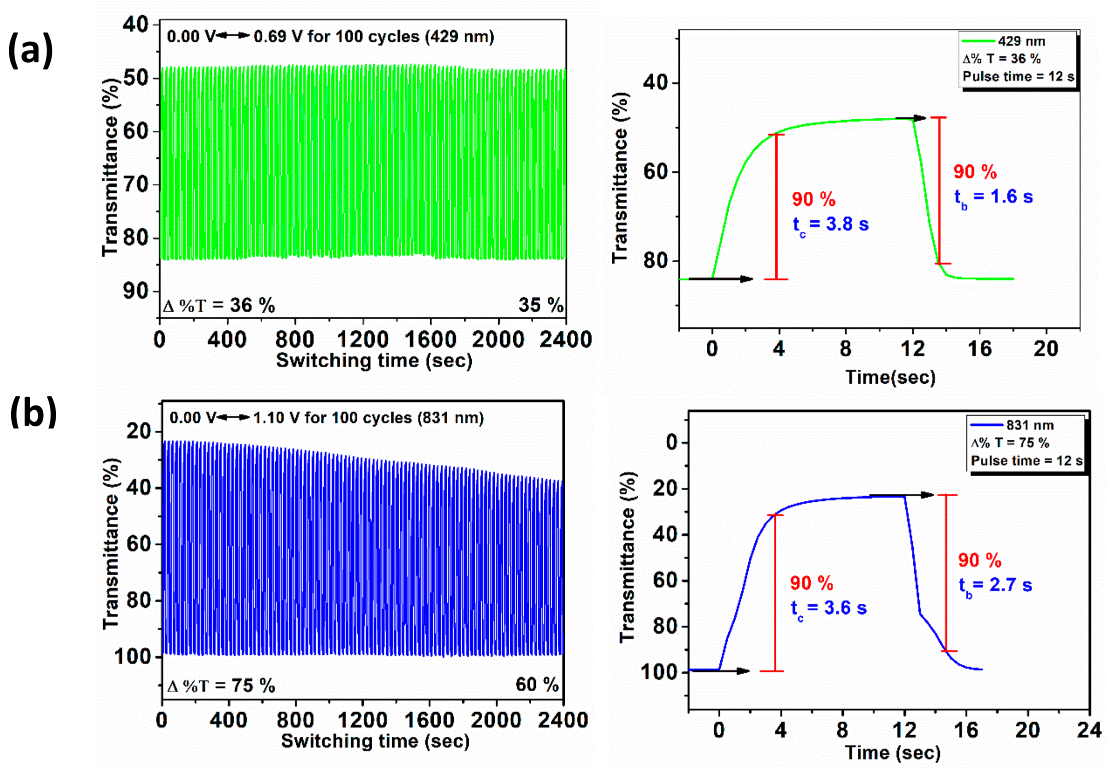

Figure S31. Electrochromic switching responses of the cast film of PAI *p*-**8e** on the ITO-glass slide (coated area ~ 0.8 × 2.2 cm^2) in 0.1 M TBAP/MeCN by applying a square-wave potential step between (a) 0.00 V and 0.69 V, monitored at λ_{max} = 429 nm and (b) 0.00 V and 1.10 V monitored at λ_{max} = 831 nm.
